# Rational design of antibodies with pH-dependent antigen-binding properties using structural insights from broadly neutralizing antibodies against α-neurotoxins

**DOI:** 10.1080/19420862.2025.2553624

**Published:** 2025-09-11

**Authors:** Jack Wade, Nina Štrancar, Monica L. Fernández-Quintero, Suzana Siebenhaar, Tom Jansen, Edward P. W. Meier, Timothy P. Jenkins, Sara P. Bjørn, Giang T. T. Nguyen, Bruno Lomonte, José Maria Gutiérrez, Christoffer V. Sørensen, Johannes R. Loeffler, Arijit Paul, Tulika Tulika, Johnny Arnsdorf, Sanne Schoffelen, Emil V. S. Lundquist, Jennifer Sørensen, Andrew B. Ward, Bjørn G. Voldborg, Markus-Frederik Bohn, Esperanza Rivera-de-Torre, J. Preben Morth, Andreas H. Laustsen

**Affiliations:** a Department of Biotechnology and Biomedicine, Technical University of Denmark, Denmark; b Center for Molecular Biosciences Innsbruck, Department of General, Inorganic and Theoretical Chemistry, University of Innsbruck, Innsbruck, Austria; c Department of Integrative Structural and Computational Biology, The Scripps Research Institute, San Diego, CA, USA; d Instituto Clodomiro Picado, Facultad de Microbiologia, Universidad de Costa Rica, San Jose, Costa Rica; e BioInnovation Institute, Copenhagen, Denmark

**Keywords:** pH-dependent antigen binding, acid-switched antibodies, snakebite, monoclonal antibodies, phage display, snake venom

## Abstract

Antibodies that bind in a pH-dependent manner to their antigens show promise for enhanced neutralization potency and blocking capacity against extracellular targets. However, because the mechanisms governing pH-dependent antigen binding remain poorly understood, engineering approaches are often limited to incorporating histidine residues in the antibody complementarity-determining regions. Here, we use a panel of human monoclonal antibodies with neutralizing activity to long-chain α-neurotoxins (LNTxs) to investigate pH-dependent antigen binding. The antibodies vary in their light chains but have conserved histidine residues in their variable domains, allowing us to explore how other residues may affect pH dependence. Comparative structural and molecular dynamics studies between two antibodies with and without pH-dependent antigen-binding properties reveal that both antibodies neutralize LNTxs by mimicking LNTx-receptor interactions through their heavy chains. We hypothesize that part of the pH-dependency can be controlled by the light chain through modulation of water access to residues at the heavy-light-chain interface. We show that pH-dependent antigen-binding properties can be introduced into monoclonal antibodies through the substitution of selected residues at the heavy-light-chain interface. Specifically, we replaced tyrosine residues in the light chain with small polar and apolar amino acid residues in a structurally related anti-LNTx antibody with limited inherent pH-dependent antigen-binding properties, and found that these smaller substitutions enhanced pH-dependence more effectively than histidine substitutions alone. Our findings suggest a strategy for engineering pH-dependent antigen binding in antibodies that goes beyond the exclusive use of histidine doping.

## GRAPHICAL ABSTRACT

**Figure:**
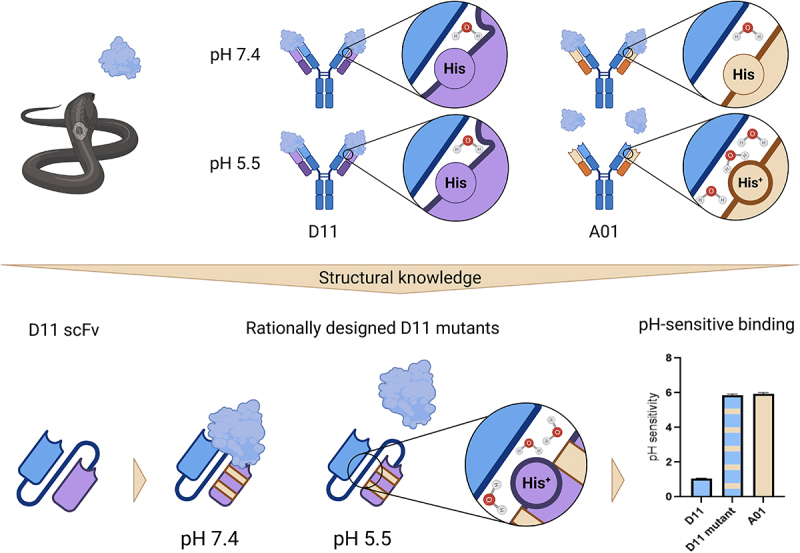

## Introduction

Precisely controlling extracellular and intracellular pH is crucial for the myriad of biochemical reactions that occur within cells. It also plays a central role in the immune response, particularly in antibody recycling within endosomes.^1^ Thus, it has increasingly become a focal point for tuning antibody specificity for therapeutic applications, such as developing monoclonal antibodies (mAbs) against viruses and diseases driven by extracellular proteins.^1,2^

**Figure 1.**
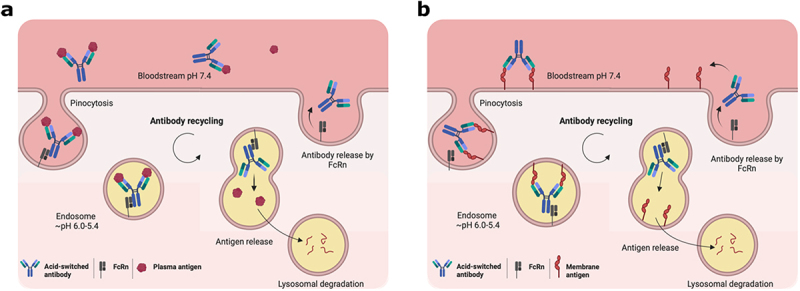
Antibodies with pH-dependent antigen binding properties. a. In plasma, antibodies with pH-dependent antigen binding properties (blue) bind antigens (red) at pH 7.4 and enter cells via pinocytosis. Upon the pH decrease in the endosome, antibodies are captured by the neonatal Fc receptor (FcRn) (grey) and release their antigens for degradation via the lysosomes, while the antibody is recycled alone into the bloodstream. b. Alternatively, antibodies binding to membrane-anchored antigens undergo receptor-mediated internalisation and are similarly recycled unbound. In both scenarios a and b, the extracellular concentration of unbound antibodies remains high for an extended period, enabling further binding to plasma antigens or reattachment to cell surfaces by binding membrane-anchored antigens.

Antibody recycling commences in the endosomes and is facilitated by the neonatal Fc receptor (FcRn), which transports antibodies from acidified endosomes back into the blood plasma.^3,4^ The interaction between FcRn and antibodies is strictly pH dependent, allowing FcRn to bind to antibodies selectively in the endosome and to rescue them from lysosomal degradation, leading to subsequent release at neutral pH at the cell surface.^3^ Recycling antibodies is an important feature in their use as therapeutics, as it increases their half-life relative to other drug classes and thereby lowers the needed dose and/or dosage frequency.^3^ It has also been exploited to expand the scope of diseases that may be treated with an antibody. For example, the change in pH during antibody recycling has been harnessed to discover and design novel antibodies with pH-dependent antigen binding. Antibodies with pH-dependent antigen binding, also known as acid-switched antibodies or recycling/sweeping antibodies, bind their antigens with high affinity at physiological pH (~7.4) until reaching the endosome.^5^ Here, upon exposure to low pH, they dissociate from their antigens and are returned unbound to the blood plasma by FcRn, as opposed to either being recycled still bound or being degraded in lysosomes along with the antigens (Figure 1). Therefore, antibodies with pH-dependent antigen binding likely interact with multiple molecules of their cognate antigens before being eliminated.^5,6^ In addition, they may have a reduced target-mediated clearance and prolonged half-life compared to conventional antibodies.^7–9^ This concept has been applied to develop acid-switched antibodies for treating rare diseases caused by dysregulation of the complement system, where the use of conventional antibodies would otherwise be impractical due to the need for excessively high or frequent dosing (ClinicalTrials.gov: NCT03406507, NCT04434092, NCT02946463).^10–12^ Acid-switched antibodies are likewise in preclinical development for treating chronic inflammatory diseases (e.g., endometriosis)^2^ and are under investigation for use against cancer. Specifically, acid-switched antibodies targeting the immune checkpoint program death receptor-ligand-1 (PD-L1) have demonstrated superior tumor regression of PD-L1 positive tumors compared to their non-pH-dependent counterparts.^13^ Finally, recent work has also shown that acid-switched antibodies can enhance the degradation activity of antibody-based lysosome-targeting chimeras (LYTACs) for the selective degradation of membrane proteins.^14^

Previous reports using structural studies of pH-dependent antibodies have established a connection with histidine residues within the paratope or the epitope.^9,13^ However, examples now exist where both the epitope of the antigen and the paratope of the antibody are devoid of histidine residues, demonstrating that other amino acid residues also contribute to pH-dependent antigen-binding properties.^15^ Antibodies with pH-dependent antigen binding have occasionally been isolated from immune repertoires,^2,7^ but they are typically developed by protein engineering to introduce pH dependence into their interaction with an antigen. As histidine residues (pK_a_ value of ~6.5 when exposed to solvents under physiological conditions) can ionize under natural conditions within the pH range that an antibody experiences during recycling, substitution of other amino acid residues with histidine residues (i.e., histidine doping) has become a common strategy for engineering pH-dependent antigen binding into antibodies.^5,6,16–21^ Identification of pH-sensitive “hot spots” for histidine residue substitutions has therefore been explored using directed evolution technologies, such as phage or yeast displays, or by single mutation scanning using automation.^4,10–12^ However, these approaches have also been used to discover antibodies whose pH-dependent antigen-binding properties either derive from or are further enhanced by other residues than histidine.^9,21,22^ Additionally, it has been found that water molecules can influence the pK_a_ values of buried residues.^23–25^ In combination, this suggests that expanding the focus beyond histidine to include other amino acid residues may enhance the discovery of antibodies with pH-dependent antigen binding, offering improved developability and therapeutic potential for *in vivo* applications.

Here, we use a panel of human mAbs containing conserved histidine residues in their variable domains as a model system to identify mutations that confer pH-dependent antigen binding. This system also serves as a platform for developing a new strategy for rational pH-engineering of antibodies. We previously developed antibodies for broad neutralization of snake venom toxins (long-chain α-neurotoxins, LNTxs) by optimizing the affinity of the parent antibody by light-chain shuffling affinity maturation.^26^ However, we did not investigate their neutralization mechanism or whether they could also bind to LNTxs in a pH-dependent manner. To establish the molecular basis for pH-dependent antigen binding in these antibodies, we determined at five different pHs the crystal structures of two neutralizing antibodies (A01 and D11) with different pH-dependent binding affinities to LNTxs from the elapids *Naja kaouthia* and *Bungarus multicinctus*. We then conducted molecular dynamics simulations to investigate how the light (V_L_) chain and alternative residues to histidines present in these antibodies control their different pH sensitivities to LNTxs. These results formed the basis for us to rationally increase the pH-sensitivity of the D11 antibody, which itself does not display pH-dependent antigen-binding properties. By particularly focusing on the use of non-histidine residue substitutions in the variable chain interface, as well as focusing on the dissociation rate between antibody and antigen as the basis for pH-dependence, we demonstrate a therapeutically relevant mechanism for introducing pH-dependency into antibodies, which may provide a broader route to optimizing antibodies for pH-dependent antigen binding.

## Results

### Identification of the cross-reactive, pH-dependent mAb A01

**Figure 2.**
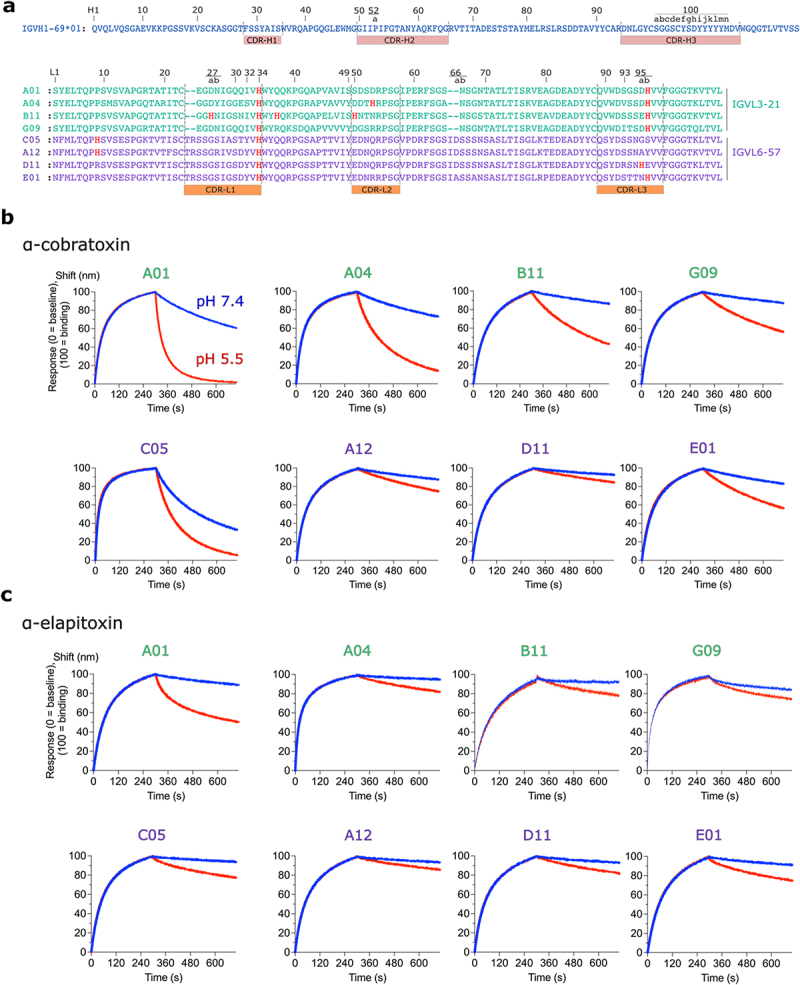
Screening of mAbs for pH-dependent binding properties to long-chain α-neurotoxins. a. Amino acid sequences of mAbs (numbered according to the kabat scheme). All mAbs share a common heavy-chain (blue) of IGHV1-69*01 germline origin, paired with either an IGLV3-21 (green) light-chain or an IGLV6-57 (purple) light-chain. Light-chain variable domain sequences are aligned. Histidine residues are coloured red. b, c. Binding of mAbs to biotinylated α-cobratoxin or α-elapitoxin captured on streptavidin-coated biolayer interferometry biosensors. The association was conducted at pH 7.4, followed by dissociation at pH 7.4 (blue) and pH 5.5 (red).

To discover antibodies that exhibit pH-dependent antigen-binding properties without the use of histidine doping, we generated a panel of mAbs with conserved histidine residues in their variable domains that are found in natural immune repertoires (Figure 2a). All mAbs share an identical heavy (V_H_) chain from the IGHV1-69*01 germline gene, paired with either an IGLV6-57 light-chain (mAbs: A12, D11, and E01) or an IGLV3-21 light-chain (mAbs: A01, A04, B11, and G09). The different light chains were introduced using light-chain shuffling on the parent mAb, 368_01_C05 (C05), which has an IGLV6-57 light-chain and was also included in the study.^27^ The light chain complementarity-determining region (CDR) sequences between individual mAbs are similar among mAbs derived from the same germline (Figure 2a). However, between germlines, there are noticeable sequence lengths and residue differences, particularly in the CDR-L1 loop of the IGLV6-57 light-chains as compared to the IGLV3–21 light-chains (Figure 2a).

To screen for pH-dependent binding, the mAbs were converted to an antigen-binding fragment (Fab) format (see Supplementary Figure S1) and assessed for pH-dependent binding to the toxins used during phage display selections: namely α-cobratoxin from *N. kaouthia* (Uniprot ID: P01391) and α-elapitoxin-Dpp2d (from hereon: α-elapitoxin) from *Dendroaspis polylepis* (Uniprot ID: C0HJD7). pH-dependent binding to the toxins was assessed by biolayer interferometry (BLI) and was measured by the change in dissociation rate of Fabs bound to α-cobratoxin or α-elapitoxin at pH 7.4 or pH 5.5. Fabs were first screened against α-cobratoxin, which led to the identification of one antibody from the IGLV3-21 germline that was particularly pH sensitive (Figure 2b). This antibody, A01, bound α-cobratoxin with an affinity (*K*_D_) of 21.0 ± 11.1 nM and dissociated 19.7 ± 9.9 times faster from α-cobratoxin at pH 5.5 compared to pH 7.4, having increased in dissociation rate from 7.5 × 10^−4^ ± 3.7 s^−1^ at pH 7.4 to 146.0 × 10^−4^ ± 6.1 s^−1^ at pH 5.5 (Figure 2b; also, see Supplementary Table S1). The A01 Fab was also the most pH-sensitive Fab to α-elapitoxin and dissociated 16.9 ± 2.8 times faster at pH 5.5 than at pH 7.4. The affinity of A01 was stronger to α-elapitoxin (*K*_D_ = 2.1 ± 0.1 nM), which can be attributed mainly to a slower dissociation rate (Figure 2b; also, see Supplementary Table S2), which reduced from 1.7 × 10^−4^ ± 0.6 s^−1^ at pH 7.4 to 29.5 × 10^−3^ ± 4.3 s^−1^ at pH 5.5. These fold differences were comparable to other reported antibodies that display pH-dependent antigen-binding properties.^13,21^ As A01 differs from the other mAbs only in its light chain, these results indicate that residues present in the light chain are the drivers of pH-dependent binding to α-cobratoxin and α-elapitoxin.

To exclude the possibility that pH-dependent antigen binding was linked to reduced stability of the A01 binding domain at pH 5.5, we measured the temperature stability of the A01 Fab at pH 7.4 and pH 5.5 using differential scanning fluorimetry (DSF). Profiling the unfolding at increasing temperatures at pH 7.4 and pH 5.5 demonstrated that A01 has equivalent stability profiles at both measured pH levels, with unfolding temperatures at pH 7.4 of 74.30 ± 0.03°C and at pH 5.5 of 73.95 ± 0.02°C (see Supplementary Table S3). Furthermore, the A01 Fab is marginally (~1°C) more stable compared to the G09 Fab (pH 7.4 = 72.79 ± 0.01°C; pH 5.5 = 73.13 ± 0.07°C), which is from the same germline and showed reduced pH-dependent antigen-binding properties, suggesting that pH-dependent antigen binding does not necessarily come with an offset in overall stability. A01 and G09 are less stable (~2°C) than both the parental C05 Fab and the chain-shuffled D11 Fab from the IGLV6–57 germline, indicating that the antibodies with IGLV6–57 light-chains are, in general, more stable. These results suggest that the pH-dependent antigen-binding mechanism linked with the A01 light-chain is not a result of pH-dependent instability.

### *A01 mAb neutralises α-cobratoxin* in vivo

**Figure 3.**
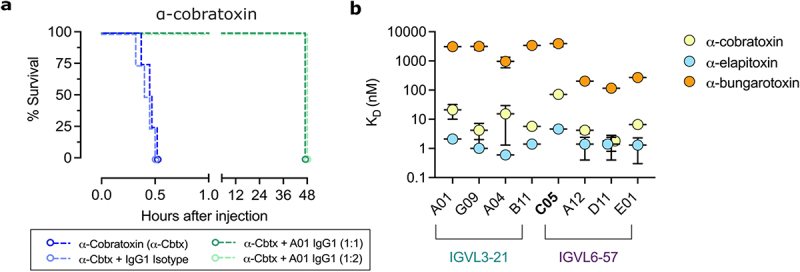
pH-dependent mAb A01 neutralises α-cobratoxin in vivo and maintains cross-reactivity to an LNTx from *bungarus multicinctus*. a. Kaplan–Meier plot showing the capacity of A01 to neutralise α-cobratoxin. A 4-µg amount of α-cobratoxin was preincubated with the A01 IgG1 at either a 1:1 or 1:2 molar ratio of toxin:igg before the mixture was injected intravenously in mice. b. BLI monovalent affinity measurements of the A01 antibody, parent antibody C05, and fellow chain-shuffled mAbs in Fab format to α-cobratoxin, α-elapitoxin, and α-bungarotoxin. *n* ≥ 2 experimental repeats and data represent the mean ± SD.

To ensure that the mAb possessed utility in a relevant disease context, we next determined whether the A01 mAb could neutralize LNTxs in vivo by preincubating 2 × LD_50_s of α-cobratoxin with different doses of A01 in IgG1 format before intravenous injection. Control mice that received α-cobratoxin only, or a preincubated mixture of a toxin and an IgG1-matched isotype control antibody, succumbed within 30 min with overt signs of neurotoxicity (i.e., limb paralysis and respiratory difficulty). In contrast, mice administered with α-cobratoxin preincubated with the A01 IgG1 in 1:1 and 1:2 toxin:IgG molar ratios all survived the recommended 24-hr observation window and did not show signs of paralysis during this time interval (Figure 3a). However, past this period, mice succumbed at some point in the following 24 hr. This result contrasts with the outcome observed in a previous study on the D11 IgG1 antibody using the same rodent model, where it provided full protection against α-cobratoxin beyond 48 hr. This observation is likely explained by the D11 mAb having a higher affinity to α-cobratoxin than A01 at pH 7.4, as reflected in the lower *K*_D_ value to α-cobratoxin as determined by BLI, primarily due to a decrease in dissociation rate (Figure 3b; also, see Supplementary Tables S1 and S2), although other pharmacokinetic or pharmacodynamic factors could also contribute to this *in vivo* observation. In addition to the differences in affinities and neutralization capacity, it was also observed that D11 and chain-shuffled mAbs with IGLV6–57 light-chains were more cross-reactive to an LNTx from *Bungarus multicinctus* elapid (α-bungarotoxin), not used during antibody discovery, than A01 and fellow mAbs with IGLV3–21 light-chains, which exhibit affinities comparable to the parental mAb, C05 (*K*_D_ ~ 3.0 µM) (Figure 3b).

### Neutralisation of LNTxs by receptor mimicry

**Figure 4.**
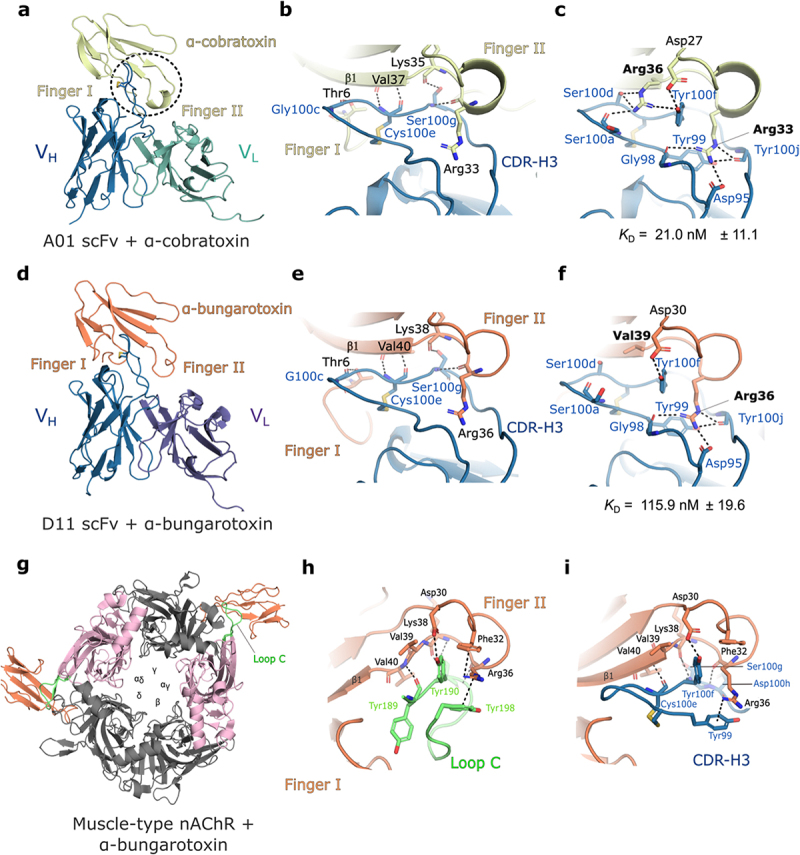
CDR-H3 contains key determinants for cross-reactivity to long-chain α-neurotoxins. a. Overall structure of the A01 scFv bound to α-cobratoxin. b. Backbone recognition of α-cobratoxin. c. Engagement of Arg residues on position 33 (Arg33) and 36 (Arg36) of α-cobratoxin at the interface between CDR-H3 and the toxin. d–f. Equivalent perspectives of the D11 scFv bound to α-bungarotoxin. Conserved V_H_ domain, blue; A01 V_L_ domain, green; α-cobratoxin, pale yellow; D11 V_L_ domain, purple; α-bungarotoxin, orange. The structurally equivalent residues are Arg36 and Val39 on α-bungarotoxin, of which Val39 results in a loss of hydrogen bonding networks with CDR-H3, corresponding with a reduction in binding affinity. Antibody residues are numbered according to the kabat scheme. g. Overview of the extracellular domains of the muscle-type nAchR from *Tetronarce californica* complexed with α-bungarotoxin (Protein Data Bank accession code, PDB: 6UWZ). The α-subunits are coloured light pink, and the interface between α-bungarotoxin and loop C in the *Tetronarce* nAchR is coloured green. h. Enlarged view of amino acid residues involved in the binding interface between α-bungarotoxin and loop C. i. The same perspective of the D11 scFv in complex with α-bungarotoxin reveals mimicry in the hydrogen bonds and cation–π interactions between loop C in the *Tetronarce* nAchR and the CDR-H3 loop.

To understand the molecular mechanism for pH-dependent antigen binding by A01, we determined the crystal structure of the A01 antibody in a complex with α-cobratoxin at different pHs (Supplementary Table S4). Accordingly, we determined the high-resolution structures of the A01 mAb produced as a single-chain variable fragment (scFv) in a complex with α-cobratoxin at pH 6.0, pH 5.5, and pH 4.5 at 1.49 Å, 1.60 Å, and 1.55 Å resolution, respectively (Supplementary Figures S2, S3; also, see Supplementary Table S4). We also sought to crystallize and determine the structure of D11 in complex with α-bungarotoxin for comparative analysis because it displays less pH-dependent antigen binding as compared to A01, and also because it is known to neutralize several LNTxs, including α-bungarotoxin from the elapid *B. multicinctus*, both *in vivo* and *in vitro*.^26^ Due to the known reactivity of the D11 mAb to α-bungarotoxin and the moderate sequence identity (SI) between α-bungarotoxin and α-cobratoxin (60% SI), we selected α-bungarotoxin for further crystallization trials at pH 6.5 and pH 7.5 with the D11 scFv (Figure 4d; Supplementary Table S4).

The total buried interface surface areas of both scFvs bound to their respective LNTx were 923.6 Å^2^ and 963.4 Å^2^ for the A01 and D11 antibody-toxin complexes, respectively, according to a Proteins, Interfaces, Structures, and Assemblies (PISA) interaction analysis.^23^ In both complexes, the heavy chain dominates the interaction, with the interface surface areas distributed in a ~ 90:10 ratio between the heavy and the light chains, respectively. The differences in the interaction surface areas are attributed mainly to the light chains, with the D11 light-chain having a larger interaction surface area than the A01 light-chain.

Both the A01 and D11 scFvs bind to a conserved conformational epitope centered on the finger II of α-cobratoxin and α-bungarotoxin through their CDR-H3 loop (Figure 4a–f). The CDR-H3 loop recognizes the backbone of finger II of both α-cobratoxin and α-bungarotoxin through a continuous hydrogen bond network with the main chain of residues on the helical loop and the β1 strand (residues: Arg33, Lys35, and Val37 in α-cobratoxin and Arg36, Lys38, and Val40 in α-bungarotoxin) (Figure 4b,e). An SGGS hairpin in the CDR-H3 protrudes between finger I and finger II of α-cobratoxin and is stabilized by a disulfide bond between Cys100^CDR-H3^ and Cys100e^CDR-H3^, facilitating a hydrogen bond between the backbone of Gly100c^CDR-H3^ in the hairpin with Thr6 in finger I. The binding is further stabilized by the formation of a short β-sheet interaction between the backbone carbonyl and amide of Cys100e^CDR-H3^ and Val37. Further analysis reveals the presence of additional hydrogen bonds, cation–π interactions, and a charged hydrogen bond (salt bridge) with the side chains of residues Arg33 and Arg36 on finger II, which occupy the center of the paratope (Figure 4c,f). Collectively, these residues are highly conserved among LNTxs (see alignment in Supplementary Figure S4).

While many of the polar contacts with α-cobratoxin and α-bungarotoxin are facilitated by ionizable residues in the antibody paratope (Tyr99^CDR-H3^, Tyr100f^CDR-H3^, Tyr100i^CDR-H3^, and Tyr100j^CDR-H3^, as well as Asp95^CDR-H3^ and Cys100e^CDR-H3^), their interactions with α-cobratoxin and α-bungarotoxin are equivalent in the bound antibody structures of A01 at pH 6.0, pH 5.5, and pH 4.5, and D11 at pH 7.5 and pH 6.5, with the exception that α-bungarotoxin has a structurally equivalent valine residue as opposed to Arg36 in α-cobratoxin (also conserved in α-elapitoxin). The valine to arginine substitution at this position results in a substantial reduction in hydrogen bonding possibilities and cation–π interactions (Figure 4c,f) and may explain the reduced affinity of all mAbs for α-bungarotoxin as compared with α-cobratoxin and α-elapitoxin, which both have Arg36, as determined by BLI (see Supplementary Table S5, S6). Finally, a structural alignment of α-bungarotoxin bound to the D11 mAb and to its target receptor, the muscle-type nicotinic acetylcholine receptor (nAChR) isolated from Pacific electric ray (*Tetronarce californica*) revealed an equivalent network of hydrogen bonds and cation–π interactions between the CDR-H3 loop and the loop C in the nAChR, mimicking the interaction with α-bungarotoxin. Particularly conspicuous interactions are Tyr99^CDR-H3^, Tyr100i^CDR-H3^, and Cys100e (backbone carbonyl), which occupy similar hydrogen bonding networks as loop C residues from nAChR Tyr189, Tyr190, and Tyr189, respectively. A similar interface is found in α-cobratoxin.

Collectively, these results provide a basis for the neutralization of LNTxs by the A01 and D11 mAbs by receptor mimicry, thereby inhibiting toxin interactions with the nAChR through the heavy-chain CDR-H3 loop. However, these results indicate no obvious effect of ionizable residues in the CDR-H3 loop interactions in facilitating pH-dependency, consistent with the fact that the CDR-H3 loop is conserved in the antibodies, despite their differences in pH-dependency.

### pH-dependency is not facilitated through histidine residues in the binding interface

**Figure 5.**
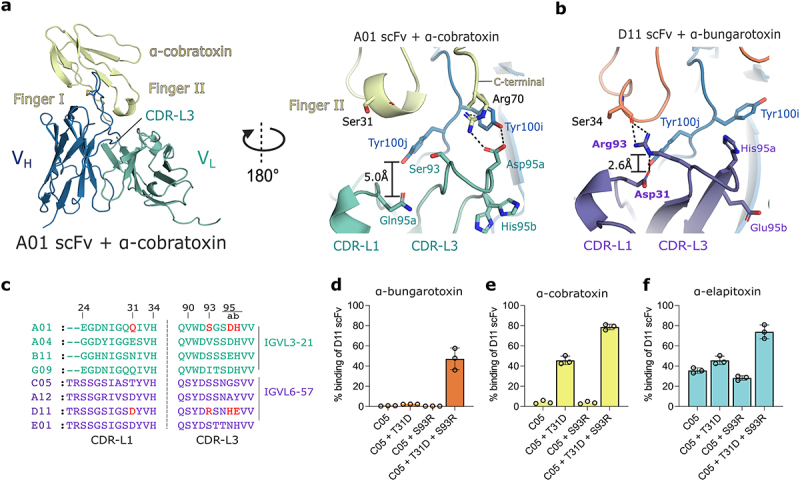
Light-chain interactions with LNTxs. a. Interactions between the A01 V_L_ and α-cobratoxin. b. Equivalent perspective of D11 V_L_ with α-bungarotoxin, highlighting differences in the interaction networks with each LNTx. Equivalent residues in the A01 and D11 scFv CDR-L3 regions are shown as sticks, hydrogen bonds between Asp31 and Arg93 are shown as dashed lines, and the equivalent interaction in A01 is shown as a double T bar c. Sequence alignment of CDR-L1 and CDR-L3 loops of the chain-shuffled mAbs and parent mAb C05. d–f. Effect of substitutions at positions of Asp31 (T31D) and Arg93 (S93R) on binding activity to α-bungarotoxin, α-cobratoxin, and α-elapitoxin, respectively. Substitutions were done in the parent mAb (C05) due to it having the same CDR-L1 loop length as D11. Data represent the mean value of four technical repeat measurements ± SD. A01 V_L_ is coloured green; D11 V_L,_ purple; and conserved V_H_, blue. α-bungarotoxin and α-cobratoxin are coloured orange and pale yellow, respectively.

The A01 and D11 mAbs mediate further polar interactions to α-cobratoxin and α-bungarotoxin with their light chains through ionizable residues in their CDR-L3 loops (Figure 5c,d). The A01 light-chain stabilizes a cation-π interaction between the side chains of Tyr100i^CDR-H3^ and Arg70 on the C-terminal tail of α-cobratoxin (Figure 5a). This interaction is facilitated by a hydrogen bond and salt-bridge interaction with the carboxyl group of Asp95a^CDR-L3^ (Figure 5b). By contrast, D11 forms a hydrogen bond with the backbone carbonyl of Ser34 on finger II of α-bungarotoxin through the side chain of Arg93^CDR-L3^ (Figure 5b). Since Arg93^CDR-L3^ in D11 and Asp95a^CDR-L3^ in A01 are substitutions from serine and glycine in the parent C05 mAb (Figure 5c), this indicates that the light chains enhanced cross-reactivity by forming new polar interactions with LNTxs and tyrosine residues in the CDR-H3 loop. Furthermore, there were no histidine residues in the binding interface between A01 and α-cobratoxin, indicating that pH-dependency is not coupled through the direct interactions of histidine residues in the paratope or epitope. While the interactions facilitated by Asp95a^CDR-L3^ and α-cobratoxin are equivalent at pH 6.0, pH 5.5, and pH 4.5 (Supplementary Figure S5), Asp95a^CDR-L3^ is present in the light chains of chain-shuffled mAbs A04 and G09 (Figure 2a). Since these antibodies bind more pH-dependently to α-cobrotoxin than antibodies A12, D11, C05, and E01 (Figure 2a), which encode alanine, histidine, glycine, and asparagine at this position, respectively, this indicates that the interactions of Asp95a^CDR-L3^ may in fact be pH-dependent and contribute to the pH-dependent binding by the A01 light-chain. However, despite encoding Asp95a^CDR-L3^, A01 is still more pH-dependent than the A04 and G09 antibodies. Therefore, the increased pH-sensitivity of A01 to LNTxs likely requires additional residues to Asp95a^CDR-L3^, possibly ionizable residues outside the binding interface.

Analysis of the histidine residue content in its variable domains revealed that A01 has two histidine residues in the CDR loops (His34^CDR-L1^ and His95b^CDR-L3^) (Figures 2a and 5c). To determine whether both histidine residues were important for facilitating pH-dependent binding in A01, we substituted both histidine residues with alanine residues. The impact of histidine mutation on pH-dependent binding was assessed in a dissociation-enhanced lanthanide fluorescence immunoassay (DELFIA) by capturing scFvs from supernatant and incubating with α-cobratoxin in a pH 7.4 buffered solution (see Supplementary Figure S2). After binding, α-cobratoxin-bound scFvs were subjected to a pH shift at either pH 7.4 or pH 5.8. The ratio of α-cobratoxin remaining bound after the pH change was then used to determine the pH-sensitivity of the interaction between the scFv and α-cobratoxin. The original A01 scFv and an scFv from the IGLV6–57 germline, D11, that was less pH-dependent than A01, were included as references, and as expected, the A01 scFv was clearly more pH sensitive than the D11 scFv, with pH 7.4/pH 5.8 ratios of ~12.0 and ~1.3, respectively. Hence, the key molecular determinants for pH-dependent binding were retained in the scFv format. The substitution of His34^CDR-L1^ to an alanine residue significantly reduced pH-dependent binding in the A01 scFv, lowering the ratio from ~12.0 to 1.6 (~7.0-fold reduction) (see Supplementary Figure S2). Additionally, substituting His95b^CDR-L3^ to an alanine residue also reduced pH-sensitivity to α-cobratoxin by ~3.0-fold, and substituting both histidine residues in A01 to alanine residues to create the variant A01-H34A-H95bA reduced pH-sensitivity further to a level comparable to that of the D11 scFv (1.3-fold). Thus, both histidine residues in the A01 CDR-L1 and CDR-L3 loops were pivotal for the pH-dependent binding to α-cobratoxin. In particular, the His34^CDR-L1^ residue is a conserved histidine residue in the light chains of both pH-dependent and pH-independent mAbs, including D11, and has the greatest effect in reducing pH-dependent binding in A01 to α-cobratoxin. However, both histidine residues were conserved in other mAbs that had a reduced pH-sensitivity to α-cobratoxin and α-elapitoxin (Figures 2a and 5c). Hence, these histidine residues alone cannot fully explain the pH-dependent binding behavior of the A01 mAb to α-cobratoxin and α-elapitoxin.

Finally, regarding cross-reactivity, it was observed that substituting both Asp31^CDR-L1^ and Arg93^CDR-L3^ residues back into the C05 parent, which was produced as an scFv, and measuring binding by DELFIA confirmed that both of these residues were critical for enhancing cross-reactivity to all three LNTxs (Figure 5d–f). While both residues in combination restored over 50% binding capacity to each LNTx, only Asp31^CDR-L1^ improved cross-reactivity as a single substitution, confirming the importance of Asp31^CDR-L1^ for enhancing cross-reactivity in IGLV6–57 light-chains. Due to the shorter CDR-L1 loop in A01 and its encoding Ser93^CDR-L3^ instead of Arg93^CDR-L3^, A01 is unable to form equivalent polar contacts with Tyr100i^CDR-H3^ and Ser34 on finger II of α-bungarotoxin, and may explain the observed increase in cross-reactivity of D11 and chain-shuffled mAbs with IGLV6–57 light-chains to α-bungarotoxin.

### The pH-dependent antigen binding properties are governed by the light chain through the variable domain interface

**Figure 6.**
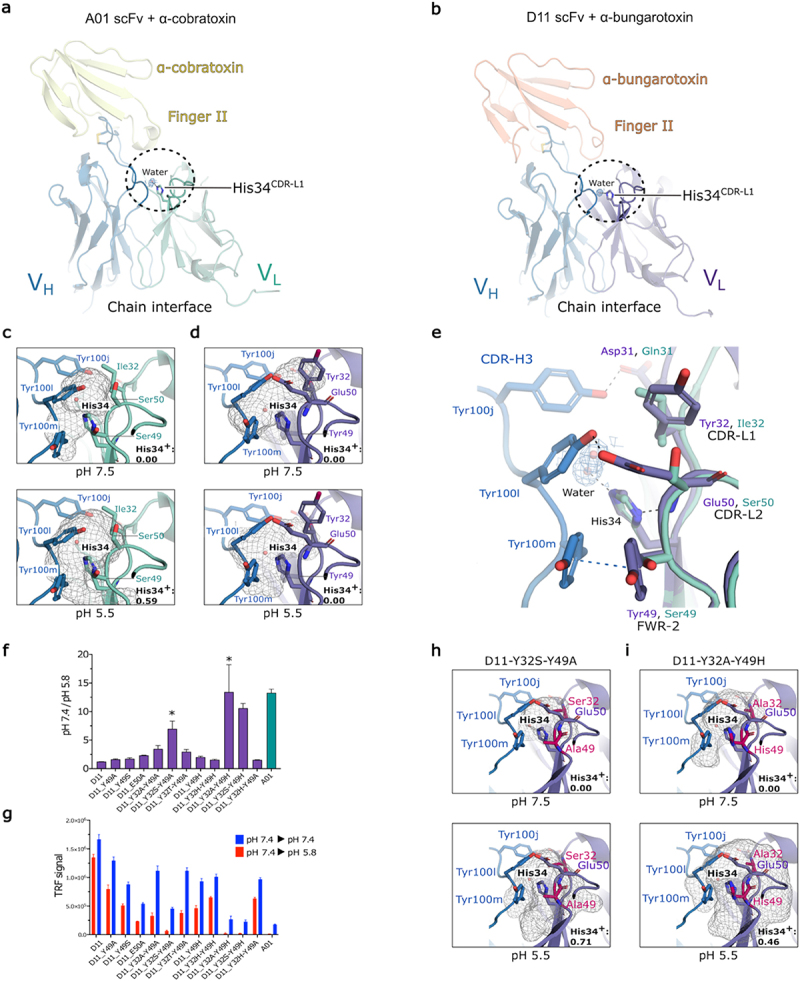
Light chains modulate water molecule access in the V_H_–V_L_ interface to control pH-dependent binding to α-cobratoxin. a, b. A cartoon representation of the A01 and D11 single-chain fragment (scFv) bound to α-cobratoxin (yellow) and α-bungarotoxin (salmon), respectively. The conserved histidine residue in the CDR-L1 loop (His34^CDR-L1^) in the chain interface is shown as a stick model, and the water molecule in red as a sphere, the V_H_ of both A01 and D11 are blue, and the V_L_ of A01 is green and D11 purple. The 2Fo-Fc electron density map surrounding the water molecule is contoured at 1 sigma and is shown as blue mesh. c, d. The grey mesh shows water probabilities around His34^CDR-L1^ from constant simulations performed on the A01 and D11 scFvs with their respective protonation probabilities at pH 7.5 and pH 5.5, in the lower right corner. The His34^CDR-L1^ residue in the A01 scFv shows an increased solvation and His34^CDR-L1^ protonation probability at pH 5.5 compared to pH 7.5 (c) as compared to D11 at an equivalent pH range (d). The side chain of Tyr49 has been removed for clarity. e. Zoom-in view of the residues involved in the V_H_–V_L_ (chain) interface for both the A01 and D11 mAbs. The A01 V_L_ domain is superimposed with the D11 V_L_ domain. Residues in the equivalent positions in the chain interfaces are shown as sticks and color coding as in a and b. f. pH-dependent binding of D11 light-chain mutant variants and the original D11 and A01 scFvs to α-cobratoxin. (*) marks variants selected for molecular dynamics simulations. g. The detection of α-cobratoxin remaining bound to scFvs after dissociation in solution buffered at pH 7.4 and pH 5.8 by time-resolved fluorescence. Data represents the average value of four technical repeat measurements ± SD. h, i. Water probabilities around His34^CDR-L1^ from molecular dynamics simulations at pH 7.5 and pH 5.5, displayed as grey mesh, of select pH-sensitive variants (Y32S-Y49A and Y32A-Y49H) of the D11 scFv at pH 7.5 and pH 5.5.

Collectively, our structural analysis indicates that pH-dependent binding to LNTxs was driven by amino acid residues in the light chain, outside the paratope–epitope interface. To further compare and investigate the light chains, we conducted molecular dynamics simulations at different pHs (pH 5.5 and pH 7.5) with the A01 and D11 scFvs bound to their respective LNTxs. Our simulations indicate that the conserved histidine residue in the CDR-L1 loop of both mAbs (His34^CDR-L1^) has a higher probability of protonation at pH 5.5 in A01 (protonation probability: 0.59) than D11 (protonation probability: ∼0.00). Protonation of His34^CDR-L1^ in A01 was accompanied by a significantly increased water molecule network around the histidine imidazole group, which enables the paratope to adopt a greater number of conformational states that are unfavorable for antigen binding at pH 5.5 compared to pH 7.5 (Figure 6a,c also, see Supplementary Figure S2). This is further quantified by a decrease in interaction energy from −9 ± 3 kcal/mol at pH 7.5 to −52 ± 15 kcal/mol at pH 5.5 of His34^CDR-L1^ with water in the V_H_–V_L_ interface (Supplementary Table S7). By contrast, His34^CDR-L1^ in D11 participates in additional interdomain interactions that may contribute to a more stabilized paratope and heavy-light-chain interface at both pH values (Figure 6b,d). The interdomain (chain interface) interactions in D11 are stabilized by hydrogen bonds and hydrophobic interactions between CDR-H3 tyrosine residues Tyr100j^CDR-H3^, Tyr100l^CDR-H3^, and Tyr100m^CDR-H3^ and light-chain residues Asp31^CDR-L1^, Tyr49^FWR-2^, and Glu50^CDR-L2^ in the neighboring protein environment of His34^CDR-L1^ (Figure 6e). The Tyr49^FWR-2^ and Glu50^CDR-L2^ residues are highly conserved in the light chains of chain-shuffled mAbs with reduced pH-sensitivity and are Ser49^FWR-2^ and Ser50^CDR-L2^, respectively, in A01. Furthermore, although not conserved in less pH-dependent mAbs, Tyr32^CDR-L1^ in D11 occupies the pocket directly above water coordinated with His34^CDR-L1^ and may shield His34^CDR-L1^ from protonation, reducing the pH-sensitivity of the antigen–antibody binding interaction. In line with these observations, we find highly similar interaction energies in the V_H_–V_L_ interface for His34^CDR-L1^at pH 7.5 and pH 5.5 (Supplementary Table S7).

We next substituted amino acid residues with either small polar or aliphatic side chains (serine, alanine, and threonine) into the D11 mAb V_L_ domain, within the vicinity of His34^CDR-L1^, and determined their effect on pH-dependent binding to α-cobratoxin in the scFv format as previously described by DELFIA and BLI (Supplementary Figure S6). In line with the differences observed in amino acid residue composition in the chain interfaces, substituting amino acid residues in D11 with similar biochemical properties to those residues found in A01 at positions Tyr32^CDR-L1^, Tyr49^FWR-2^, and Glu50^CDR-L2^ increased the pH-dependent antigen-binding properties of D11 to α-cobratoxin (Figure 6f). The variants D11-Y32A-Y49A and D11-Y32S-Y49A, designed to allow water molecules into the chain interface by substituting both Tyr32^CDR-L1^ and Tyr49^FWR-2^ residues with serine and alanine residues, respectively, released between 3.5- and 7.0-fold more toxin at pH 5.8 compared to at pH 7.4, and showed a *k*_off_ 5.5/7.4-fold difference of 3.5 and 4.7, respectively (see Supplementary Table S8).

To test whether increasing the number of histidine residues in the chain interface environment would increase pH-dependent binding to α-cobratoxin further, we designed variants with increasing histidine residue content. Directly substituting both Tyr32^CDR-L1^ and Tyr49^FWR-2^ residues with histidine to create a histidine-restricted variant (D11-Y32H-Y49H) only marginally increased pH-dependent antigen binding (fold-difference = ~1.6) compared to the original D11 mAb (fold-difference ~1.2) and did not increase pH-dependent antigen binding (k_off_ 5.5/7.4-fold difference ~1), as measured by BLI (Supplementary Figure S7). By contrast, exchanging an alanine or serine amino acid at position His32^CDR-L1^ in the D11-Y32H-Y49H variant elevated the pH-dependent binding of D11 to α-cobratoxin to a fold-difference level comparable to A01 (fold-difference ~13.4). Finally, by comparing binding signals at both pH levels between variants designed without additional histidine residue substitutions in their sequence with the D11-Y32H-Y49H variant, we identified one design (D11-Y32A-Y49A) that had comparable binding signals at pH 7.4 (Figure 6g), but it displayed approximately 2-fold increase in pH-dependent binding to α-cobratoxin (fold difference ~3.5). Similar to the findings for A01, molecular dynamics simulations indicate that the substitutions to tyrosine residues Tyr32^CDR-L1^ and Tyr49^FWR-2^ resulted in an increase in the protonation probability and solvation of His34^CDR-L1^ in D11 at pH 5.5 (Figure 6h,i), suggesting that a similar mechanism drove pH-dependent antigen-binding properties. This is also further supported by a substantially decreased interaction energy of His34^CDR-L1^ with water in the V_H_–V_L_ interface (Supplementary Table S7). Collectively, these results support that the light chains coordinate pH-dependent antigen binding by controlling the solvent accessibility and protonation state of histidine residues in the variable domain interface.

To further validate the pH-dependent binding mechanism observed through molecular dynamics simulations and BLI, we performed isothermal titration calorimetry (ITC) for A01, D11, D11-Y32H-Y49H, and D11-Y32A-Y49H at pH 7.4 and 5.5. In line with previous observations, ITC revealed that both D11 and D11-Y32H-Y49H exhibited similar thermodynamic profiles at the two pH values, indicating minimal pH-sensitivity. By contrast, A01 and D11-Y32A-Y49H showed elevated dissociation constants (*K*_D_) at pH 5.5 compared to pH 7.4, consistent with a reduced affinity toward the α-cobratoxin under acidic conditions (Supplementary Figure S8). These findings are consistent with the results obtained from other kinetic (BLI) and equilibrium (DELFIA) techniques, as well as with the pH-dependent conformational dynamics observed in molecular dynamics simulations (Supplementary Figure S9). Moreover, introducing residues other than histidine – particularly amino acids with small polar or aliphatic side chains – proved more effective at enhancing the pH-dependent binding of the D11 antibody than histidine substitutions alone.

## Discussion

Antibodies with pH-dependent antigen-binding properties can be used to develop effective therapies against a range of diseases by taking advantage of either their potential antigen sweeping properties or their ability to bind targets only below or above a specific pH.^24^ However, their molecular mechanisms are often overlooked during drug development, and therefore, they typically require both rational and random mutageneses for further development. This is usually performed by introducing histidine residues into the CDR loops at random positions.^5^ Here, we investigated the ability of our earlier reported anti-LNTx mAbs, developed to possess broad cross-reactivity and neutralization potency against LNTxs by light-chain shuffling,^26^ to engage with their targets in a pH-dependent manner. Structural analysis of two representative chain-shuffled mAbs (A01 and D11) revealed a common toxin neutralization mechanism – mimicry of the toxin interactions with loop C in the nAChR using their CDR-H3 loop. This finding is corroborated by other broadly-neutralizing human mAbs and nanobodies developed recently against LNTxs, which also derive such capacity through mimicking loop C in the receptor.^28–30^ However, in contrast to other reported antibodies with pH-dependent antigen-binding properties that are specific to different target classes, such as antibodies to the PD-1 ligand and C5 protein,^9,13^ the pH-dependency of A01 does not derive from histidine residues in the binding interface with the target, and represents a mechanism distinct from these antibodies in facilitating pH-dependent binding.

By selecting mAbs that have conserved histidine residues in their variable domains, we were able to investigate the role of alternative residues to histidine in enabling pH-dependent antigen binding. Kinetic analysis at neutral and acidic pH revealed that antibodies with conserved histidine residues in their variable domains had notable differences in their ability to dissociate from LNTxs at acidic pH (Figure 2a,b). The dissociation rate change of one antibody (A01) allowed the majority of α-cobratoxin and α-elapitoxin molecules to be released within a 5-min dissociation window at pH 5.5 (Figure 2b,c), which is in line with our recent findings that A01 can release α-cobratoxin during antibody recycling by human FcRn *in vitro*.^31^ This contrasts with the related non-pH-dependent D11 mAb, which contains the same number of histidine residues in its variable domain as A01, yet remains bound to α-cobratoxin after cellular recycling.^31^ This observation suggests that simply introducing histidine residues may be insufficient to achieve optimal pH-dependent antigen binding. We therefore hypothesize that careful tuning of antibody affinity is also critical to ensure that the antibody operates within the dynamic range necessary for efficient cellular recycling and transport.

A common feature of acid-switched or sweeping antibodies is their ability to bind and/or neutralize their target antigen at neutral pH, until the antigen is released during the FcRn-mediated recycling process.^2,20^ Although A01 has a faster dissociation rate from α-cobratoxin at pH 5.5 compared with D11, the dissociation rate is also rather fast at pH 7.4 (A01 *k*_dis_: (7.5 ± 3.7) × 10^−4^ s^−1^; D11 *k*_dis_: (1.2 ± 0.1) × 10^−4^ s^−1^). This may have contributed to the reduced *in vivo* protection of A01 by allowing α-cobratoxin molecules to be released into the extracellular environment prior to recycling, thereby allowing toxins to bind and inhibit nAChRs. Notably, the dissociation rates between both mAbs and α-elapitoxin at pH 7.4 (A01 *k*_dis_: (1.7 ± 0.6) × 10^−4^ s^−1^; D11 *k*_dis_: (1.5 ± 0.3) × 10^−4^ s^−1^), as well as their respective binding affinities, were comparable, indicating that the pH-dependent binding mechanism of A01 can be coupled with the high-affinity interactions necessary for toxin neutralization.

While the utility of broadly-neutralizing antibodies and nanobodies for the development of recombinant antivenoms against snakebite envenoming is well established,^27–30,32^ the value of having such antibodies with pH-dependent antigen-binding properties remains unknown. Addressing this question will require further investigation in appropriate preclinical models, such as rescue experiments in mice transgenic for the human FcRn receptor. Nevertheless, it can be speculated that, owing to the “depot effect” (i.e., prolonged retention of venom toxins at the bite site with slow release into circulation) observed in many snakebites, acid‑switched antibodies may also have therapeutic potential in the treatment of snakebite envenoming.^15,31^ More broadly, the findings from this study may be relevant to other indications, such as chronic diseases characterized by high expression of endogenous targets, in which prolonged neutralization by acid‑switched antibodies could provide meaningful therapeutic advantages.^31,33^

In summary, the presented structural data, combined with molecular dynamics simulations, elucidate the molecular mechanisms for neutralization and pH-dependent binding to LNTxs for a panel of mAbs. Our results demonstrate that modulating solvent access to protonatable residues in the variable domain interface can be a relevant approach for fine-tuning pH-dependence in neutralizing antibodies to LNTxs. The insights gained from this work may be particularly applicable for engineering antibodies with binding determined mainly by the heavy chain, such as bispecific antibodies designed with a common light chain,^21^ and more broadly for the design of antibodies with pH-dependent binding properties. In particular, our study highlights that acid-switched antibodies can be developed without the use of histidine doping. These findings therefore expand the toolbox for engineering pH‑dependent antigen‑binding properties into mAbs by demonstrating that mutations outside the classical CDRs can modulate the heavy-light chain interface to function as a conformational pH switch. This, in turn, may enable the use of allosteric regulation and cooperative binding as viable strategies for designing acid-switched antibodies.

## Materials and methods

### Antibody discovery

The antibodies in this study were previously discovered using a phage display-based approach involving light-chain shuffling for affinity maturation and cross-panning against α-cobratoxin from *N. kaouthia* (Uniprot ID: P01391) and α-elapitoxin-Dpp2d from *D. polylepis* (Uniprot ID: C0HJD7), as previously described.^26,34,35^ These discovery campaigns used the IONTAS phage display library, a human antibody phage display library of 4 × 10^10^ clones, with antibodies in the form of scFvs, which was constructed from B lymphocytes collected from 43 non-immunized human donors.^36^

### Antibody and Fab expression and purification

The A01 antibody and control antibodies for mouse studies were provided by Line Ledsgaard and Aneesh Karatt-Vellatt as full-length, human IgG1 isotype antibodies, produced by transient transfection using mammalian cells as previously described.^27^

For Fab production, the V_H_ and V_L_ domains were inserted into pcDNA2.0-based plasmids encoding the constant regions of the heavy and light chains. Fabs were expressed in Expi Chinese Hamster Ovary cells (ExpiCHO^TM^) (ThermoFisher Scientific, A29133) by transfecting the expression plasmid into ExpiCHO^TM^ cells using ExpiFectamine prepared in OptiPRO SFM according to the manufacturer’s guidelines. Cultures were incubated at 37°C, 130 rpm, and 8% CO_2_ under humidity-controlled conditions. The day after transfection, the enhancer was added to the cultures, and cells were allowed 168 hr to express Fabs. Secreted Fabs were harvested and purified using a HisTrap column followed by size-exclusion chromatography using a Superdex75 Increase 10/300 column. All antibody sequences were assigned using the ImMunoGeneTics information system (IMGT) database.^37^ All Fabs were analyzed by SDS-PAGE followed by Coomassie staining to estimate protein purity with and without reducing agent (see Supplementary Figure S1a,b). For the C05 Fab, multiple bands were observed and confirmed to be due to an N-linked glycosylation site in the CDR-L3 loop at position Asn95 (see Supplementary Figure S1a). For biophysical characterization, the glycosylation site was removed by substituting aspartate with a serine residue, chosen due to being the most frequently observed residue in this position in the other anti-LNTx Fabs. Subsequent analysis by SDS-PAGE confirmed the putative glycan site was removed by observing clear bands corresponding to the heavy and light chains, respectively (see Supplementary Figure S1a). For experimental repeat measurements in BLI assays, Fabs produced in a previous production using human embryonic kidney cells were included.^22,37^

### Toxin preparation

α-Cobratoxin (Latoxan, L8114) and α-bungarotoxin (Latoxan, L8115) were purchased as lyophilized purified proteins. α-Elapitoxin was isolated from the whole venom of *D. polylepis* (Latoxan, L1309) by using a C18 column (250 × 4.6 mm, 5 μm particle; Teknokroma) and established protocols.^38^ All three LNTxs were biotinylated through amine coupling chemistry using an EZ-Scientific™ NHS-PEG_4_-Biotin, No-Link™ Format, reagent (Thermo Scientific, A39259).

### pH-dependent antigen binding testing by BLI

pH-dependent binding assays with Fabs or scFvs were performed using an Octet RED96 system (ForteBio) in a running buffer of 10 mM HEPES, 50 mM MES, 150 mM NaCl, and 0.05% P20 at pH 7.4 (HEPES-MES). A system temperature at 24°C and a shake speed at 1000 rpm were used throughout the assay. Streptavidin biosensors (Sartorius, 18–5136) were equilibrated in kinetics buffer (Sartorius, 18–1105), prepared in phosphate-buffered saline (PBS: 137 mM NaCl, 3 mM KCl, 8 mM Na_2_HPO_4_·2 H_2_O, and 1.4 mM KH_2_PO_4_ at pH 7.4) in the dark for 10 min. Biotinylated LNTxs were also prepared in the kinetics buffer by diluting them to a concentration of 0.4 μg/mL. Next, streptavidin biosensor tips were loaded with biotinylated LNTxs by transferring them to wells with prepared LNTx, resulting in a surface capture level between 0.2 nm and 0.5 nm. Biosensors loaded with the LNTx were equilibrated by dipping them into wells containing HEPES-MES running buffer for 30 s and then dipped into wells containing a concentration series of Fabs or scFvs at 700 nM for 120 s to 300 s to allow for association. Biosensors with LNTx-Fab complexes were then dissociated in a HEPES-MES solution buffered to either pH 7.4 or pH 5.5 for 1000 s. The remaining Fabs or scFvs were removed from the biosensors by dipping biosensors iteratively for 10 s between wells containing 10 mM glycine and 2 M NaCl at pH 2.0 and kinetics buffer seven times. A blank biosensor without any LNTx coating was subjected to the same cycles as biosensors loaded with the LNTx and included as a reference biosensor. Data were processed in the Octet evaluation software (version 12.2.2.4). The reference was subtracted from the binding curves, and curves were fitted using a 1:1 binding model with a global fit. The *K*_D_ values were determined as a product of the kinetic rates (*k*_d_/*k*_a_) and pH-dependency by the change in dissociation rate between pH 7.4 and pH 5.5 (*k*_d pH 5.5_ /*k*_d pH 7.4_). For weaker interactions with α-bungarotoxin, the affinity was measured under steady-state conditions using a one-site specific model.

### Isothermal titration calorimetry

The binding affinities between α-cobratoxin and the A01 scFv, as well as the D11 scFv and its selected mutants, were analyzed by ITC, using a Nano ITC instrument (TA instruments) at 25°C. Proteins were dialyzed (Thermo Scientific Slide-A-Lyzer Dialysis Cassettes, 3.5 K MWCO, 3 mL 10759784) against 5 L of sterile PBS at pH 7.4 or 5.5. Thereafter, protein concentrations were determined using the theoretical molar extinction coefficients calculated based on the amino acid content using the Expasy ProtParam tool. The toxin was loaded into the cell and titrated with the A01 scFv as well as D11 scFv and its selected mutants, and the toxin was diluted to 10 μM in the cell and the scFvs to 100 μM in the syringe. The titrations were carried out at 25°C, starting with an injection of 5.0 μL followed by 13 injections of 7.0 μL (subsequently spaced by 600 s between the injections). A blank correction for the heat of dilution was subtracted in all experiments performed. A single set of equivalent and independent binding site models was fit to the resulting binding isotherms, which allowed for the determination of the equilibrium association constant (*K*a), the binding stoichiometry (n), the molar-binding enthalpy (ΔH), and the derived calculation of free Gibbs Energy (ΔG), and entropy (ΔS).

### Thermal unfolding of Fabs

The melting temperature (T_m_) of the Fabs was assessed using the Nanotemper Prometheus Panta system. Fabs were desalted using 7 kDa MWCO cartridges (Thermo Scientific 89,890) and prepared at a concentration >0.5 mg/mL in a HEPES-MES buffer. Samples were then loaded into capillaries provided by NanoTemper Technologies and subjected to a gradual temperature increase from 15°C to 95°C, with a thermal ramping rate of 1°C/min. Fluorescence readings at 330 nm and 350 nm were recorded to determine the T_m_. Analysis of the data was conducted using PR Panta Analysis v.1.4.3 software.

### Animals

*In vivo* assays were conducted in CD-1 mice of both sexes of 18–20 g body weight, supplied by Instituto Clodomiro Picado, following protocols approved by the Institutional Committee for the Use and Care of Animals (CICUA), University of Costa Rica (approval number CICUA 82-08). Mice were housed in Tecniplast Eurostandard Type II 1264C cages in groups of four animals per cage and were provided food and water *ad libitum* and maintained at 18–24°C, 60–65% relative humidity, and 12:12-hr light–dark cycles.

### In vivo *preincubation experiments*

The *in vivo* neutralizing potential of antibody A01 against α-cobratoxin from the venom of *N. kaouthia* was assessed. The antibody was incubated with α-cobratoxin at toxin:antibody molar ratios of 1:1 and 1:2. Controls included α-cobratoxin incubated with either 0.12 M NaCl and 0.04 M phosphates at pH 7.2 (PBS) or with an isotype control IgG. After incubation, aliquots of 100 µL, containing 4 µg α-cobratoxin, corresponding to two median lethal doses (LD_50_), were injected intravenously, in the caudal vein, into groups of four mice. Following injection, animals were observed for signs of neurotoxicity, and survival was monitored for 48 hr. Deaths were recorded, and Kaplan–Meier plots were used to represent mouse survival over time.

### Expression and purification of scFv proteins

The microbial expression vector encoding the variable domain sequences of the A01 and D11 antibodies fused covalently by a serine-glycine linker and containing a C-terminal 6xHis-FLAG extension, was constructed previously.^29^ For structural studies, a TEV endoprotease cleavage site was inserted immediately after the C-terminus of the V_L_ domain (TVLENLYFQSGQPAAASAHHHHHHKLDYKDHDGDYKD), such that the cleavage site (underlined) preceded the 6xHis-FLAG extension using *in vivo* assembly cloning.^26,27^ To express the scFvs, *Escherichia coli* BL21(DE3) bacterial cells (New England Biolabs, C2527H) harboring an expression plasmid were autoinduced and incubated overnight as described previously,^27,39^ with the exception that Tunair™ shake flasks were used to increase expression. Affinity purification of scFvs was carried out using a HisTrap™ HP 5 mL purification column (Cytiva, 17–5255–01) and buffer exchanged into 20 mM Tris, 50 mM NaCl, and 5 mM EDTA at pH 8.0 buffer using PD-10 columns (Merck, GE17–0851–01). To obtain untagged scFvs, the C-terminal 6xHis-FLAG was removed by incubation overnight with SuperTEV endoprotease, which was added at a 20-fold lower molarity than the scFv. The scFvs were purified by size-exclusion chromatography using a Superdex 75 10/60 HiLoad column (Cytiva 28,989,333), which was run using 5 mM Tris, 20 mM NaCl, and pH 8.0 (TN) buffer as eluent at 4°C. Eluted fractions that contained the monomeric scFv with the C-terminal tag removed, as judged by SDS-PAGE, were pooled and the sample stored at 4°C. The sample was used within a week for crystallography.

### Crystallisation and structure determination

Lyophilized α-cobratoxin (Latoxan, L8114) and α-bungarotoxin (Latoxan, L8115) were redissolved in the TN buffer to a concentration of 5 mg/mL. Freshly prepared A01 and D11 scFvs were concentrated to 20 mg/mL using 3 kDa ultracentrifugation spin concentration units (Thermo Scientific™, 88525). The LNTxs and scFvs were allowed to be complex overnight at 4°C in a 1:3 scFv:LNTx ratio. The scFv-LNTx complexes were isolated in the TN buffer at 4°C using a Superdex 75 10/30 GL column (Sigma-Aldrich, GE17–5174–01), and both complex partners were confirmed to coelute by SDS-PAGE (see Supplementary Figure S3a-d). The individual scFv-LNTx complexes were concentrated above 12 mg/mL using protein concentrator spin columns (Sartorius, VN01H91), and crystallization trials were performed at 18°C by the sitting-drop vapor diffusion method using a mosquito robot (mosquito Xtal3, SPT Labtech). For the D11 scFv-α-bungarotoxin complex, crystals grew within 12 hr in the Molecular Dimensions MCSG2 crystallization screen plate (condition: 0.1 M MES/NaOH, 12% PEG 20,000, pH 6.5 and 0.1 M HEPES, pH 7.5, 70% (v/v) MPD). For the A01 scFv in complex with α-cobratoxin, crystals appeared within 1 week. After optimization, the first crystals appeared after 2 weeks in 1.2 μL drops prepared in a ratio of 2:1 protein complex to reservoir solution. The crystals were developed as multilayered plates at different pHs, and the highest-resolution data were collected from drops containing the following conditions: 0.25 M bis-tris, pH 4.5, 0.3 M ammonium sulfate, 24% PEG 3350; 0.1 M bis-tris pH 5.5, 0.25 M ammonium sulfate, 25% PEG3350; and 0.1 M bis-tris pH 6.0, 0.3 M ammonium sulfate, 25% PEG3350.

The A01-α-cobratoxin crystals were flash-frozen by immersion into a cryo-protectant containing the crystallization solution supplemented with a final concentration of 20% glycerol (v/v).

Diffraction data were obtained at the P14 EMBL (PETRA III, Hamburg, Germany) and Biomax (MAX IV Laboratory, Lund, Sweden) beamlines. The complete data set was processed from 140° (1400 images) for the A01 scFv bound to α-cobratoxin and 360° (3600 images) for the D11 scFv bound to α-bungarotoxin. Both A01 and D11 bound to their respective LNTxs exhibited P2_1_2_1_2_1_ space group symmetry and had two copies of the scFV in the complex with the LNTx in the asymmetric unit. The data were processed with Xia2^40^ with XDS.^41^ The crystal structure of the A01 scFv bound to α-cobratoxin was determined by molecular replacement with Phaser-MR^37^ from the PHENIX suite^42^ using an AlphaFold2^43^ as a template search model built from the A01 scFv sequence and a crystal structure of the toxin (PDB: 4AEA). The final models were built in Coot^44^ with refinement performed with phenix.refine.^42^ The co-crystal between the D11 scFv and α-bungarotoxin was determined using an equivalent workflow but used the solved co-crystal of the A01 scFv structure and α-bungarotoxin (PDB: 6UWZ) as search models. The structures were evaluated using MolProbity, with final statistics presented in Supplementary Table S1. The final models contain the variable domain sequences of both antibodies and the primary sequence of LNTxs with two disordered regions: the Ser-Gly linker between the scFv V_H_ and V_L_ domains and amino acid residues 73 and 74 at the C-terminal of α-bungarotoxin, respectively. Molecular graphics were presented with PyMOL Molecular Graphics System (Version 2.2r7pre, Schrödinger, LLC).

### Dissociation-enhanced lanthanide fluorescence assays for scFv-LNTx binding

Anti-FLAG M2 antibody (Sigma Aldrich, F1804) was diluted in a PBS buffer to a concentration of 2.5 μg/mL and captured onto black 96-well MaxiSorp Immuno plates (Thermo Scientific 437,111) overnight at 4°C using a 50 μL volume. Plates were washed three times with PBS, and the wells were blocked by adding 200 μL of a blocking/binding buffer consisting of 3% nonfat dried milk powder (VWR, A0830.1000) prepared in PBS (MPBS) and incubated for 1 hr at room temperature with 250 rpm shaking. The scFv molecules were produced following established protocols^39,45^ and harvested by centrifugation for 15 min at 4000 g. Plates were washed three times with PBS and exposed to scFv supernatants, diluted 2-fold in 6% MPBS, and left for 1 hr. Plates were subsequently washed three times with PBS supplemented with 0.1% Tween 20 (PBS-T) and three times with PBS. Biotinylated LNTxs were prepared in MPBS to a concentration that was close to the experimentally determined *K*_D_ values of the original wildtype antibodies, as determined by BLI using Fab formats. For binding to α-cobratoxin and α-elapitoxin, 50 μL of 50 nM biotinylated toxins in MPBS was added to the captured scFvs, whereas 100 nM was used for α-bungarotoxin. Due to sample constraints, higher concentrations of biotinylated α-bungarotoxin could not be tested. Plates were washed three times with PBS-T and PBS prior to dissociating LNTx-bound scFvs in 200 μL MPBS (pH 7.4 or pH 5.8). Wells subjected to dissociation at pH 5.8 were primed in the final wash step by washing with PBS adjusted to pH 5.8 before adding MPBS at pH 5.8. A lower pH could not be tested due to the stability of the milk. For wells with pH 7.4 dissociation, the plates were washed three times with PBS-T and PBS before adding MPBS at pH 7.4. After washing the wells three times with PBS-T and PBS, streptavidin conjugated with Europium (Perkin Elmer, 1244–360) was prepared by diluting 1:500 times in the DELFIA assay buffer (Perkin Elmer, 1244–111), and 50 μL was added to each well for 30 min. Plates were then washed with PBS-T and PBS followed by the addition of 50 μL of DELFIA enhancement solution (Perkin Elmer, 4001–0010), which was incubated for 15 min with 250 rpm shaking. Time-resolved fluorescence (TRF) signal at excitation and emission wavelengths of 320 nm and 615 nm was measured using a plate reader (Victor Nivo, Perkin Elmer) at 25°C. The pH-sensitivity of scFvs binding to LNTxs was quantified by calculating the pH 7.4:pH 5.8 TRF signal ratios. To compare scFvs with respect to pH 7.4 binding, the same assay was applied but without a dissociation step at either pH 7.4 or pH 5.8.

### Molecular dynamics simulations protocol

Based on the crystal structure of the A01 and D11 antibodies in complex with α-cobratoxin and α-bungarotoxin, respectively, a previously published simulation protocol was used to characterize the CDR loop ensembles in solution^46,47^ to elucidate the influence of protonation on the resulting conformational states/diversity of the Fv. The starting structures for the simulations were prepared in MOE (Molecular Operating Environment 2022.02; Chemical Computing Group ULC).^48^

### Constant pH simulations and molecular dynamics simulation protocol

To identify the most probable protonation states of the antibodies with and without the antigen present at pH 7.5, pH 6.0, and pH 5.5, we performed each three 100-ns of constant pH simulations using the implementation of explicit solvent in AMBER by Roitberg and coworkers.^49^ In this constant pH approach, the simulation is interrupted at periodic intervals, and protonation changes are attempted based on a Monte Carlo Metropolis criterion. To neutralize the charges, the uniform background charge was used, which is required to compute long-range electrostatic interactions.^50^ Using the tleap tool of the AmberTools20^51^ package, the structures were soaked in cubic water boxes of TIP3P water molecules with a minimum wall distance of 12 Å to the protein.^47,52^ For all simulations, parameters of the AMBER force field 14SB were used.^51^ To enhance the sampling of the conformational space, well-tempered bias-exchange metadynamics^53,54^ simulations were performed in GROMACS^55–57^ with the PLUMED 2 implementation.^57^ Metadynamics was chosen as the simulation approach, since it enhances sampling on predefined collective variables (CVs). The sampling is accelerated by a history-dependent bias potential, which is constructed in the space of the CVs. As CVs, a well-established protocol, boosting a linear combination of sine and cosine of the ψ torsion angles of all six CDR loops calculated with functions MATHEVAL and COMBINE implemented in PLUMED 2, was used.^57^ As discussed previously, the ψ torsion angle captures conformational transitions comprehensively.^52^ The underlying method presented here has been validated in various studies against experimental results^47,58^ to be as close to the experimental conditions as possible and to obtain the correct density distributions of both protein and water. A Gaussian height of 10.0 kJ/mol was employed. Gaussian deposition occurred every 5000 steps, and a bias factor of 10 was used. Bias-exchange metadynamics simulations were performed for 500 ns for the prepared Fv structures. The resulting trajectories were aligned to the whole Fv and clustered with cpptraj^59^ using the average linkage hierarchical clustering algorithm with a root mean square deviation cutoff criterion of 1.2 Å, resulting in a large number of clusters. The cluster representatives for the antibody fragments were equilibrated and simulated for 100 ns using the AMBER 20 simulation package.

Molecular dynamics simulations were performed in an NpT ensemble using pmemd.cuda.^60^ Bonds involving hydrogen atoms were restrained by applying the SHAKE algorithm,^61^ allowing a time step of 2 fs. Atmospheric pressure of the system was preserved by weak coupling to an external bath using the Berendsen algorithm.^62^ The Langevin thermostat was used to maintain the temperature during simulations at 300 K.^63,64^ In total, 25.8 µs of simulation time for the antibody A01 at pH 7.5 and 38.9 µs at pH 5.5 were accumulated.

With the obtained trajectories, a time-lagged independent component analysis (tICA) using the Python library PyEMMA 2, employing a lag time of 15 ns, was performed. tICA was applied to identify the slowest movements of the investigated Fv fragments and consequently to obtain a kinetic discretization of the sampled conformational space.^59,65^ tICA is a dimensionality reduction technique that detects the slowest-relaxing degrees of freedom and facilitates kinetic clustering, which is a crucial prerequisite for building a Markov state model (MSM).^66^ It linearly transforms a set of high-dimensional input coordinates to a set of output coordinates by finding a subspace of “good reaction coordinates.” Thereby, tICA finds coordinates of maximal autocorrelation at a given lag time. The lag time sets a lower limit to the timescales considered in the tICA and the MSM. Accordingly, tIC1 and tIC2 represent the two slowest degrees of freedom of the systems.

Based on the tICA conformational spaces, thermodynamics and kinetics were calculated with an MSM^67^ using PyEMMA 2, which uses the *k*-means clustering algorithm to define microstates and the PCCA+ clustering algorithm^68^ to coarse-grain the microstates into macrostates. MSMs are network models that provide valuable insights into conformational states and transition probabilities between them, as they allow for the identification of boundaries between two states.^65,67^ Basically, MSMs coarse-grain the conformational diversity of the system, which reflects the free energy surface and ultimately determines the structure and dynamics of the system. Thus, MSMs provide important insights and enhance the understanding of states and transition probabilities, facilitating a quantitative connection with experimental data.^69^

The sampling efficiency and the reliability of the MSM (e.g., defining optimal feature mappings) have been evaluated with the Chapman-Kolmogorov test by using the variational approach for Markov processes and monitoring the fraction of states used, since the network states must be fully connected to calculate probabilities of transitions and the relative equilibrium probabilities. To build the MSM, the backbone torsions of the respective CDR loops were used; 150 microstates using the *k*-means clustering algorithm were defined, and a lag time of 25 ns was applied.

For the complexes of A01 and D11 and D11 mutants with the respective toxins at pH 7.5 and pH 5.5, three repetitions of 1 µs of classical molecular dynamics simulations were performed following the aforementioned simulation protocol. For wild type D11, we also modeled the complex with α-cobratoxin. The interaction energies were calculated with cpptraj using the linear interaction energy tool.^70^ The electrostatic and van der Waals interaction energies were calculated for all frames of each simulation and provided the simulation-averages of these interactions.

### Generation of A01 and D11 scFv variants

Variants of either the parental C05 mAb or chain-shuffled mAbs carrying individual point mutations in the light chain were generated using in vivo assembly cloning. Briefly, forward oligonucleotides complementary to the V_L_ gene, precloned as an scFv into the pSANG10-3F microbial expression vector, and encoding a point mutation, along with reverse oligonucleotides, were ordered from TAG Copenhagen. The forward and reverse oligonucleotides were designed with a 12–15 bp 5’ and 3’ homology to enable homologous recombination in bacteria. The plasmid was amplified using the Phusion High-Fidelity polymerase enzyme (New England Biolabs) and the template was removed by incubation with DpnI (Thermo Scientific, FD1703) for 1 hr at 37°C. The resulting PCR amplicons containing the plasmid backbone and the D11 scFv harboring a point mutation were then transformed into XL1-Blue MR supercompetent cells (Agilent Technologies 200,229) according to the manufacturer’s guidelines. Individual colonies containing the desired substitution were verified by Sanger sequencing, and the sequenced plasmids were then transformed into BL21(DE3) cells (New England Biolabs, C2527H) for expression and subsequent assessment of pH-dependent binding activity by DELFIA.

## Supplementary Material

D1292144249valreportfullP1.pdf

D1292139978valreportfullP1.pdf

Wade et al 2025 Supporting Information Revision 1.pdf

D1292144274valreportfullP19HUO.pdf

D1292139980valreportfullP1.pdf

D1292144272valreportfullP19HXO.pdf

## Data Availability

All models and structure factors are deposited into PDB under the accession codes 9FYT, 9HUO, and 9HXO (A01 bound to α-cobratoxin at pH 4.5, 5.5, and 6.5, respectively) and 9FYS and 9HUB (D11 bound to α-bungarotoxin at pH 6.5 and 7.5, respectively).
