# Supplementary material for: Rational design of antibodies with pH-dependent antigen-binding properties using structural insights from broadly neutralizing antibodies against α-neurotoxins
Source: MAbs. 2025 Sep 11;17(1):2553624. doi: 10.1080/19420862.2025.2553624 (PMC12439581; doi:10.1080/19420862.2025.2553624)
Supplement: D1292139978valreportfullP1.pdf [file KMAB_A_2553624_SM1041.pdf]

Full wwPDB X-ray Structure Validation Report (i)

Jul 4, 2024 – 11:23 am BST

PDB ID	:	9FYT
Title	:	mAbs in complex with cobratoxin at pH 4.5
Deposited on	:	2024-07-03
Resolution	:	1.55 Å(reported)

This wwPDB validation report is for manuscript review

This is a Full wwPDB X-ray Structure Validation Report.

This report is produced by the wwPDB biocuration pipeline after annotation of the structure.

We welcome your comments at *validation@mail.wwpdb.org*
A user guide is available at
https://www.wwpdb.org/validation/2017/XrayValidationReportHelp
with specific help available everywhere you see the (i) symbol.

The types of validation reports are described at
http://www.wwpdb.org/validation/2017/FAQs#types.

The following versions of software and data (see references (i)) were used in the production of this report:

MolProbity	:	4.02b-467
Mogul	:	1.8.4, CSD as541be (2020)
Xtriage (Phenix)	:	1.13
EDS	:	2.37.1
Percentile statistics	:	20191225.v01 (using entries in the PDB archive December 25th 2019)
Refmac	:	5.8.0158
CCP4	:	7.0.044 (Gargrove)
Ideal geometry (proteins)	:	Engh & Huber (2001)
Ideal geometry (DNA, RNA)	:	Parkinson et al. (1996)
Validation Pipeline (wwPDB-VP)	:	2.37.1

For Manuscript Review

1 Overall quality at a glance (i)

The following experimental techniques were used to determine the structure:
X-RAY DIFFRACTION

The reported resolution of this entry is 1.55 Å.

Percentile scores (ranging between 0-100) for global validation metrics of the entry are shown in the following graphic. The table shows the number of entries on which the scores are based.

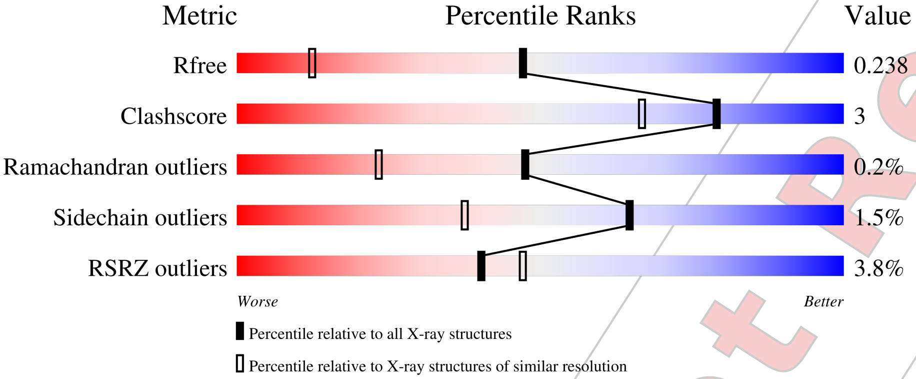

Metric	Whole archive (#Entries)	Similar resolution (#Entries, resolution range(Å))
R_{free}	130704	1483 (1.56-1.56)
Clashscore	141614	1529 (1.56-1.56)
Ramachandran outliers	138981	1498 (1.56-1.56)
Sidechain outliers	138945	1495 (1.56-1.56)
RSRZ outliers	127900	1465 (1.56-1.56)

The table below summarises the geometric issues observed across the polymeric chains and their fit to the electron density. The red, orange, yellow and green segments of the lower bar indicate the fraction of residues that contain outliers for >=3, 2, 1 and 0 types of geometric quality criteria respectively. A grey segment represents the fraction of residues that are not modelled. The numeric value for each fraction is indicated below the corresponding segment, with a dot representing fractions <=5% The upper red bar (where present) indicates the fraction of residues that have poor fit to the electron density. The numeric value is given above the bar.

Mol	Chain	Length	Quality of chain
1	A	71	3% / 93% 7%
1	B	71	7% / 94% 6%
2	C	144	5% / 80% 10% • 9%
2	I	144	4% / 83% 7% • 9%

Continued on next page...

For Manuscript Review

Continued from previous page...

Mol	Chain	Length	Quality of chain
3	D	117	% 91% 7% •
3	M	117	3% 94% • •

For Manuscript Review

2 Entry composition (i)

There are 8 unique types of molecules in this entry. The entry contains 10132 atoms, of which 4673 are hydrogens and 0 are deuteriums.

In the tables below, the ZeroOcc column contains the number of atoms modelled with zero occupancy, the AltConf column contains the number of residues with at least one atom in alternate conformation and the Trace column contains the number of residues modelled with at most 2 atoms.

- Molecule 1 is a protein called Alpha-cobratoxin.

Mol	Chain	Residues	Atoms						ZeroOcc	AltConf	Trace
			Total	C	H	N	O	S			
1	A	71	1065	332	524	98	101	10	0	0	0
1	B	71	1065	332	524	98	101	10	0	0	0

- Molecule 2 is a protein called Light chain scFv A01.

Mol	Chain	Residues	Atoms						ZeroOcc	AltConf	Trace
			Total	C	H	N	O	S			
2	I	131	2031	655	990	173	204	9	0	7	0
2	C	131	1981	640	965	166	201	9	0	3	0

- Molecule 3 is a protein called heavy chain scFv Ao1.

Mol	Chain	Residues	Atoms						ZeroOcc	AltConf	Trace
			Total	C	H	N	O	S			
3	M	114	1681	539	810	148	182	2	0	4	0
3	D	114	1709	546	823	151	187	2	0	5	0

- Molecule 4 is CHLORIDE ION (three-letter code: CL) (formula: Cl).

Mol	Chain	Residues	Atoms		ZeroOcc	AltConf
			Total	Cl		
4	A	3	4	4	0	1

- Molecule 5 is GLYCEROL (three-letter code: GOL) (formula: $C_3H_8O_3$).

For Manuscript Review

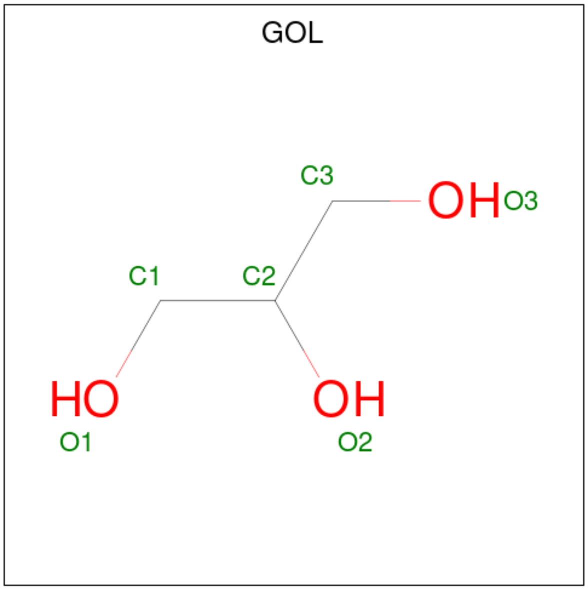

Mol	Chain	Residues	Atoms	ZeroOcc	AltConf
5	B	1	Total C H O<br>13 3 7 3	0	0
5	B	1	Total C H O<br>14 3 8 3	0	0
5	M	1	Total C H O<br>14 3 8 3	0	0
5	D	1	Total C H O<br>13 3 7 3	0	0
5	D	1	Total C H O<br>13 3 7 3	0	0

- Molecule 6 is SULFATE ION (three-letter code: SO4) (formula: O_4S).

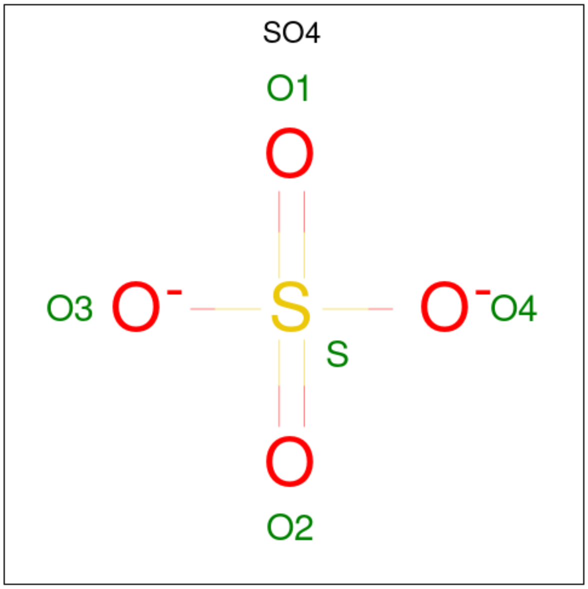

For Manuscript Review

Mol	Chain	Residues	Atoms	ZeroOcc	AltConf
6	I	1	Total O S<br>5 4 1	0	0

- Molecule 7 is SODIUM ION (three-letter code: NA) (formula: Na).

Mol	Chain	Residues	Atoms	ZeroOcc	AltConf
7	M	1	Total Na<br>1 1	0	0
7	D	1	Total Na<br>1 1	0	0

- Molecule 8 is water.

Mol	Chain	Residues	Atoms	ZeroOcc	AltConf
8	A	59	Total O<br>59 59	0	0
8	B	60	Total O<br>60 60	0	0
8	I	98	Total O<br>98 98	0	0
8	M	107	Total O<br>108 108	0	1
8	C	87	Total O<br>87 87	0	0
8	D	110	Total O<br>110 110	0	0

For Manuscript Review

3 Residue-property plots (i)

These plots are drawn for all protein, RNA, DNA and oligosaccharide chains in the entry. The first graphic for a chain summarises the proportions of the various outlier classes displayed in the second graphic. The second graphic shows the sequence view annotated by issues in geometry and electron density. Residues are color-coded according to the number of geometric quality criteria for which they contain at least one outlier: green = 0, yellow = 1, orange = 2 and red = 3 or more. A red dot above a residue indicates a poor fit to the electron density (RSRZ > 2). Stretches of 2 or more consecutive residues without any outlier are shown as a green connector. Residues present in the sample, but not in the model, are shown in grey.

- Molecule 1: Alpha-cobratoxin

Chain A:

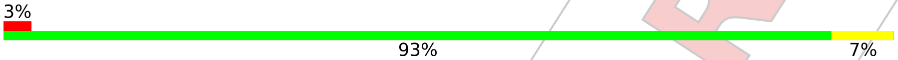

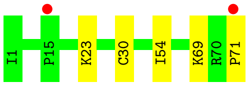

- Molecule 1: Alpha-cobratoxin

Chain B:

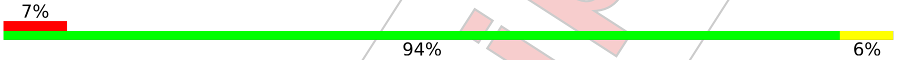

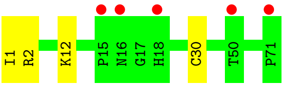

- Molecule 2: Light chain scFv A01

Chain I:

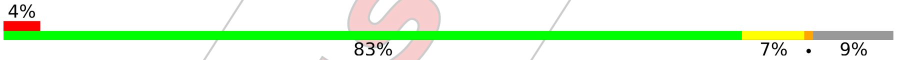

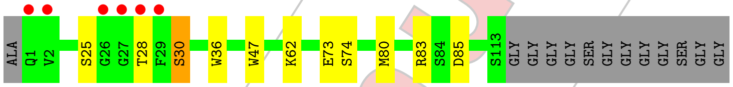

- Molecule 2: Light chain scFv A01

Chain C:

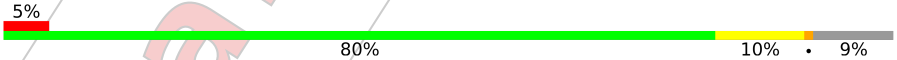

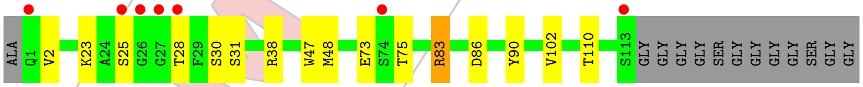

- Molecule 3: heavy chain scFv Ao1

Chain M:

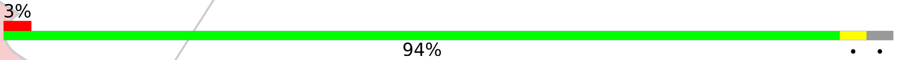

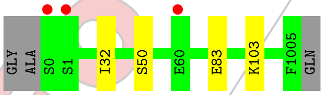

- Molecule 3: heavy chain scFv Ao1

For Manuscript Review

Chain D:

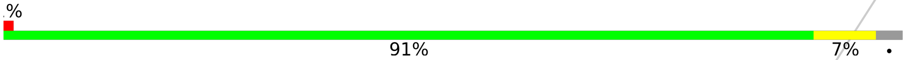

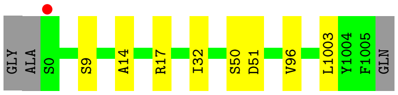

For Manuscript Review

4 Data and refinement statistics (i)

Property	Value	Source
Space group	P 21 21 21	Depositor
Cell constants<br>a, b, c, α, β, γ	76.86Å 83.86Å 98.65Å<br>90.00° 90.00° 90.00°	Depositor
Resolution (Å)	42.52 – 1.55<br>42.52 – 1.55	Depositor<br>EDS
% Data completeness<br>(in resolution range)	99.3 (42.52-1.55)<br>99.3 (42.52-1.55)	Depositor<br>EDS
R_{merge}	(Not available)	Depositor
R_{sym}	(Not available)	Depositor
$< I/\sigma(I) >$ [1]	1.32 (at 1.55Å)	Xtriage
Refinement program	PHENIX 1.20.1_4487	Depositor
R, R_{free}	0.185 , 0.237<br>0.188 , 0.238	Depositor<br>DCC
R_{free} test set	4584 reflections (4.96%)	wwPDB-VP
Wilson B-factor (Å^2)	21.5	Xtriage
Anisotropy	0.594	Xtriage
Bulk solvent k_{sol}(e/Å^3), B_{sol}(Å^2)	0.38 , 40.2	EDS
L-test for twinning[2]	$< \|L\| > = 0.49$, $< L^2 > = 0.32$	Xtriage
Estimated twinning fraction	No twinning to report.	Xtriage
F_o,F_c correlation	0.97	EDS
Total number of atoms	10132	wwPDB-VP
Average B, all atoms (Å^2)	36.0	wwPDB-VP

Xtriage's analysis on translational NCS is as follows: *The analyses of the Patterson function reveals a significant off-origin peak that is 56.54 % of the origin peak, indicating pseudo-translational symmetry. The chance of finding a peak of this or larger height randomly in a structure without pseudo-translational symmetry is equal to 2.6985e-05. The detected translational NCS is most likely also responsible for the elevated intensity ratio.*

[1] Intensities estimated from amplitudes.
[2] Theoretical values of $< |L| >$, $< L^2 >$ for acentric reflections are 0.5, 0.333 respectively for untwinned datasets, and 0.375, 0.2 for perfectly twinned datasets.

For Manuscript Review

5 Model quality (i)

5.1 Standard geometry (i)

Bond lengths and bond angles in the following residue types are not validated in this section: GOL, SO4, CL, NA

The Z score for a bond length (or angle) is the number of standard deviations the observed value is removed from the expected value. A bond length (or angle) with $|Z| > 5$ is considered an outlier worth inspection. RMSZ is the root-mean-square of all Z scores of the bond lengths (or angles).

Mol	Chain	Bond lengths		Bond angles	
		RMSZ	#$\|Z\|$ >5	RMSZ	#$\|Z\|$ >5
1	A	0.49	0/553	0.72	0/750
1	B	0.50	0/553	0.73	0/750
2	C	0.54	1/1043 (0.1%)	0.70	0/1412
2	I	0.54	0/1084	0.72	0/1466
3	D	0.50	0/917	0.65	0/1253
3	M	0.48	0/910	0.66	0/1244
All	All	0.51	1/5060 (0.0%)	0.69	0/6875

All (1) bond length outliers are listed below:

Mol	Chain	Res	Type	Atoms	Z	Observed(Å)	Ideal(Å)
2	C	90	TYR	CE2-CZ	-5.61	1.31	1.38

There are no bond angle outliers.

There are no chirality outliers.

There are no planarity outliers.

5.2 Too-close contacts (i)

In the following table, the Non-H and H(model) columns list the number of non-hydrogen atoms and hydrogen atoms in the chain respectively. The H(added) column lists the number of hydrogen atoms added and optimized by MolProbity. The Clashes column lists the number of clashes within the asymmetric unit, whereas Symm-Clashes lists symmetry-related clashes.

Mol	Chain	Non-H	H(model)	H(added)	Clashes	Symm-Clashes
1	A	541	524	524	3	0
1	B	541	524	524	4	0
2	C	1016	965	962	7	0
2	I	1041	990	984	7	0

Continued on next page...

For Manuscript Review

Continued from previous page...

Mol	Chain	Non-H	H(model)	H(added)	Clashes	Symm-Clashes
3	D	886	823	819	6	0
3	M	871	810	796	2	0
4	A	4	0	0	0	0
5	B	12	15	16	2	0
5	D	12	14	16	0	0
5	M	6	8	8	0	0
6	I	5	0	0	0	0
7	D	1	0	0	0	0
7	M	1	0	0	0	0
8	A	59	0	0	0	0
8	B	60	0	0	1	0
8	C	87	0	0	0	0
8	D	110	0	0	0	0
8	I	98	0	0	3	0
8	M	108	0	0	0	0
All	All	5459	4673	4649	27	0

The all-atom clashscore is defined as the number of clashes found per 1000 atoms (including hydrogen atoms). The all-atom clashscore for this structure is 3.

All (27) close contacts within the same asymmetric unit are listed below, sorted by their clash magnitude.

Atom-1	Atom-2	Interatomic distance (Å)	Clash overlap (Å)
2:I:30:SER:OG	2:I:73:GLU:OE2	1.68	1.11
2:I:83[B]:ARG:NH2	8:I:301:HOH:O	2.13	0.81
3:D:14:ALA:HB3	3:D:17:ARG:HG3	1.86	0.57
2:C:30:SER:OG	2:C:73:GLU:OE2	2.20	0.54
1:B:1:ILE:N	5:B:101:GOL:H2	2.26	0.50
2:C:2:VAL:HG13	2:C:102:VAL:HG21	1.95	0.49
3:M:83:GLU:HG2	3:M:103:LYS:NZ	2.29	0.47
3:D:32:ILE:CG2	3:D:50:SER:HA	2.45	0.47
3:D:50:SER:O	3:D:51:ASP:HB2	2.15	0.46
2:I:36:TRP:CE2	2:I:80[B]:MET:HB3	2.50	0.46
2:C:23:LYS:HE3	2:C:75:THR:O	2.16	0.46
2:I:80[B]:MET:O	2:I:80[B]:MET:HG3	2.17	0.45
1:B:2:ARG:NH2	8:B:203:HOH:O	2.51	0.44
2:I:25:SER:HB3	8:I:327:HOH:O	2.19	0.43
2:C:28:THR:OG1	2:C:31:SER:HB3	2.18	0.43
3:D:14:ALA:HB1	3:D:1003:LEU:HD23	2.00	0.43
1:A:69:LYS:O	1:A:71:PRO:HD3	2.19	0.43
1:A:23:LYS:HG2	1:A:54:ILE:HD12	1.99	0.43

Continued on next page...

For Manuscript Review

Continued from previous page...

Atom-1	Atom-2	Interatomic distance (Å)	Clash overlap (Å)
2:C:38:ARG:HD3	2:C:48[A]:MET:SD	2.59	0.42
3:D:32:ILE:HG23	3:D:50:SER:HA	2.01	0.42
1:A:30:CYS:HB3	1:B:30:CYS:HB3	2.02	0.42
3:M:32:ILE:CG2	3:M:50:SER:HA	2.50	0.41
2:I:85:ASP:OD2	8:I:302:HOH:O	2.22	0.41
2:C:47:TRP:CG	3:D:96:VAL:HB	2.55	0.41
2:C:83:ARG:HD2	2:C:86:ASP:OD2	2.20	0.41
1:B:1:ILE:H2	5:B:101:GOL:H2	1.87	0.40

There are no symmetry-related clashes.

5.3 Torsion angles ⓘ

5.3.1 Protein backbone ⓘ

In the following table, the Percentiles column shows the percent Ramachandran outliers of the chain as a percentile score with respect to all X-ray entries followed by that with respect to entries of similar resolution.

The Analysed column shows the number of residues for which the backbone conformation was analysed, and the total number of residues.

Mol	Chain	Analysed	Favoured	Allowed	Outliers	Percentiles	
1	A	69/71 (97%)	66 (96%)	3 (4%)	0	100	100
1	B	69/71 (97%)	66 (96%)	3 (4%)	0	100	100
2	C	132/144 (92%)	128 (97%)	4 (3%)	0	100	100
2	I	136/144 (94%)	129 (95%)	6 (4%)	1 (1%)	22	5
3	D	117/117 (100%)	112 (96%)	5 (4%)	0	100	100
3	M	116/117 (99%)	111 (96%)	5 (4%)	0	100	100
All	All	639/664 (96%)	612 (96%)	26 (4%)	1 (0%)	47	23

All (1) Ramachandran outliers are listed below:

Mol	Chain	Res	Type
2	I	28	THR

For Manuscript Review

5.3.2 Protein sidechains (i)

In the following table, the Percentiles column shows the percent sidechain outliers of the chain as a percentile score with respect to all X-ray entries followed by that with respect to entries of similar resolution.

The Analysed column shows the number of residues for which the sidechain conformation was analysed, and the total number of residues.

Mol	Chain	Analysed	Rotameric	Outliers	Percentiles	
1	A	64/64 (100%)	64 (100%)	0	100	100
1	B	64/64 (100%)	63 (98%)	1 (2%)	62	35
2	C	108/108 (100%)	105 (97%)	3 (3%)	43	14
2	I	112/108 (104%)	108 (96%)	4 (4%)	35	8
3	D	100/96 (104%)	99 (99%)	1 (1%)	76	57
3	M	99/96 (103%)	99 (100%)	0	100	100
All	All	547/536 (102%)	538 (98%)	9 (2%)	65	35

All (9) residues with a non-rotameric sidechain are listed below:

Mol	Chain	Res	Type
1	B	12	LYS
2	I	30	SER
2	I	62[A]	LYS
2	I	62[C]	LYS
2	I	74	SER
2	C	25	SER
2	C	83	ARG
2	C	110	THR
3	D	9	SER

Sometimes sidechains can be flipped to improve hydrogen bonding and reduce clashes. All (2) such sidechains are listed below:

Mol	Chain	Res	Type
1	A	16	ASN
2	C	61	GLN

5.3.3 RNA (i)

There are no RNA molecules in this entry.

For Manuscript Review

5.4 Non-standard residues in protein, DNA, RNA chains (i)

There are no non-standard protein/DNA/RNA residues in this entry.

5.5 Carbohydrates (i)

There are no monosaccharides in this entry.

5.6 Ligand geometry (i)

Of 12 ligands modelled in this entry, 6 are monoatomic - leaving 6 for Mogul analysis.

In the following table, the Counts columns list the number of bonds (or angles) for which Mogul statistics could be retrieved, the number of bonds (or angles) that are observed in the model and the number of bonds (or angles) that are defined in the Chemical Component Dictionary. The Link column lists molecule types, if any, to which the group is linked. The Z score for a bond length (or angle) is the number of standard deviations the observed value is removed from the expected value. A bond length (or angle) with $|Z| > 2$ is considered an outlier worth inspection. RMSZ is the root-mean-square of all Z scores of the bond lengths (or angles).

Mol	Type	Chain	Res	Link	Bond lengths			Bond angles		
					Counts	RMSZ	$\#\|Z\| > 2$	Counts	RMSZ	$\#\|Z\| > 2$
5	GOL	D	1102	-	5,5,5	0.72	0	5,5,5	1.23	0
5	GOL	B	101	-	5,5,5	0.82	0	5,5,5	0.86	0
6	SO4	I	201	-	4,4,4	0.16	0	6,6,6	0.36	0
5	GOL	B	102	-	5,5,5	0.85	0	5,5,5	0.89	0
5	GOL	D	1101	-	5,5,5	0.86	0	5,5,5	1.19	1 (20%)
5	GOL	M	1101	-	5,5,5	0.71	0	5,5,5	0.98	0

In the following table, the Chirals column lists the number of chiral outliers, the number of chiral centers analysed, the number of these observed in the model and the number defined in the Chemical Component Dictionary. Similar counts are reported in the Torsion and Rings columns. '-' means no outliers of that kind were identified.

Mol	Type	Chain	Res	Link	Chirals	Torsions	Rings
5	GOL	D	1102	-	-	2/4/4/4	-
5	GOL	B	101	-	-	1/4/4/4	-
5	GOL	B	102	-	-	4/4/4/4	-
5	GOL	D	1101	-	-	1/4/4/4	-
5	GOL	M	1101	-	-	1/4/4/4	-

There are no bond length outliers.

All (1) bond angle outliers are listed below:

For Manuscript Review

Mol	Chain	Res	Type	Atoms	Z	Observed(^{o})	Ideal(^{o})
5	D	1101	GOL	C3-C2-C1	-2.20	103.16	111.70

There are no chirality outliers.

All (9) torsion outliers are listed below:

Mol	Chain	Res	Type	Atoms
5	B	102	GOL	O1-C1-C2-C3
5	D	1102	GOL	C1-C2-C3-O3
5	D	1102	GOL	O2-C2-C3-O3
5	B	102	GOL	C1-C2-C3-O3
5	B	102	GOL	O2-C2-C3-O3
5	B	102	GOL	O1-C1-C2-O2
5	B	101	GOL	O1-C1-C2-C3
5	M	1101	GOL	C1-C2-C3-O3
5	D	1101	GOL	O1-C1-C2-C3

There are no ring outliers.

1 monomer is involved in 2 short contacts:

Mol	Chain	Res	Type	Clashes	Symm-Clashes
5	B	101	GOL	2	0

5.7 Other polymers (i)

There are no such residues in this entry.

5.8 Polymer linkage issues (i)

There are no chain breaks in this entry.

For Manuscript Review

6 Fit of model and data (i)

6.1 Protein, DNA and RNA chains (i)

In the following table, the column labelled '#RSRZ> 2' contains the number (and percentage) of RSRZ outliers, followed by percent RSRZ outliers for the chain as percentile scores relative to all X-ray entries and entries of similar resolution. The OWAB column contains the minimum, median, 95th percentile and maximum values of the occupancy-weighted average B-factor per residue. The column labelled 'Q< 0.9' lists the number of (and percentage) of residues with an average occupancy less than 0.9.

Mol	Chain	Analysed	<RSRZ>	#RSRZ>2			OWAB(Å^2)	Q<0.9
1	A	71/71 (100%)	0.07	2 (2%)	53	60	22, 31, 49, 75	0
1	B	71/71 (100%)	0.30	5 (7%)	16	19	25, 33, 57, 77	0
2	C	131/144 (90%)	0.41	7 (5%)	26	31	20, 30, 59, 87	0
2	I	131/144 (90%)	0.31	6 (4%)	32	37	20, 29, 50, 75	0
3	D	114/117 (97%)	-0.03	1 (0%)	84	87	22, 30, 47, 87	0
3	M	114/117 (97%)	0.03	3 (2%)	56	63	22, 30, 47, 83	0
All	All	632/664 (95%)	0.19	24 (3%)	40	47	20, 31, 53, 87	0

All (24) RSRZ outliers are listed below:

Mol	Chain	Res	Type	RSRZ
2	C	27	GLY	18.0
2	I	26	GLY	9.4
2	I	27	GLY	7.9
2	C	26	GLY	6.9
2	C	28	THR	6.6
2	I	28	THR	5.4
1	B	71	PRO	5.1
3	M	0	SER	4.6
1	A	71	PRO	3.5
2	C	1	GLN	3.5
3	D	0	SER	3.3
2	C	25	SER	3.3
3	M	60	GLU	2.7
1	A	15	PRO	2.6
2	I	1	GLN	2.5
1	B	15	PRO	2.5
1	B	16	ASN	2.4
2	I	2	VAL	2.4
2	C	113	SER	2.3

Continued on next page...

For Manuscript Review

Continued from previous page...

Mol	Chain	Res	Type	RSRZ
2	C	74	SER	2.3
1	B	18	HIS	2.3
1	B	50	THR	2.2
2	I	29	PHE	2.2
3	M	1	SER	2.0

6.2 Non-standard residues in protein, DNA, RNA chains (i)

There are no non-standard protein/DNA/RNA residues in this entry.

6.3 Carbohydrates (i)

There are no monosaccharides in this entry.

6.4 Ligands (i)

In the following table, the Atoms column lists the number of modelled atoms in the group and the number defined in the chemical component dictionary. The B-factors column lists the minimum, median, 95th percentile and maximum values of B factors of atoms in the group. The column labelled 'Q< 0.9' lists the number of atoms with occupancy less than 0.9.

Mol	Type	Chain	Res	Atoms	RSCC	RSR	B-factors($Å^2$)	Q<0.9
5	GOL	D	1102	6/6	0.71	0.18	44,89,113,136	0
4	CL	A	103[B]	1/1	0.78	0.20	44,44,44,44	1
4	CL	A	103[A]	1/1	0.78	0.20	65,65,65,65	1
5	GOL	B	102	6/6	0.80	0.23	42,96,146,146	0
5	GOL	D	1101	6/6	0.81	0.18	40,82,97,100	0
4	CL	A	102	1/1	0.85	0.30	93,93,93,93	0
5	GOL	M	1101	6/6	0.88	0.14	40,68,125,125	0
7	NA	M	1102	1/1	0.90	0.12	41,41,41,41	0
5	GOL	B	101	6/6	0.91	0.13	31,40,97,116	0
4	CL	A	101	1/1	0.91	0.20	65,65,65,65	0
6	SO4	I	201	5/5	0.96	0.11	32,38,44,53	0
7	NA	D	1103	1/1	0.98	0.10	36,36,36,36	0

6.5 Other polymers (i)

There are no such residues in this entry.

For Manuscript Review
