# Supplementary material for: Rational design of antibodies with pH-dependent antigen-binding properties using structural insights from broadly neutralizing antibodies against α-neurotoxins
Source: MAbs. 2025 Sep 11;17(1):2553624. doi: 10.1080/19420862.2025.2553624 (PMC12439581; doi:10.1080/19420862.2025.2553624)
Supplement: Wade et al 2025 Supporting Information Revision 1.pdf [file KMAB_A_2553624_SM1040.pdf]

Rational design of antibodies with pH-dependent antigen binding properties using structural insights from broadly-neutralizing antibodies against α-neurotoxins

Jack Wade[1], Nina Štrancar[1], Monica L. Fernández-Quintero[2,3], Suzana Siebenhaar[1], Tom Jansen[1], Edward P. W. Meier[1], Timothy P. Jenkins[1], Sara P. Bjørn [1], Giang T. T. Nguyen[1], Bruno Lomonte[4], José Maria Gutiérrez[4], Christoffer V. Sørensen[1,5], Johannes R. Loeffler[2,3], Arijit Paul[1], Tulika Tulika[1], Johnny Arnsdorf[1], Sanne Schoffelen [1], Emil V. S. Lundquist[1], Jennifer Sørensen[1], Andrew B. Ward[3], Bjørn G. Voldborg[1], Markus-Frederik Bohn[1], Esperanza Rivera-de-Torre[1]*, J. Preben Morth[1]*, Andreas H. Laustsen[1]*

[1]Department of Biotechnology and Biomedicine, Technical University of Denmark, Kongens Lyngby, Denmark
[2]Center for Molecular Biosciences Innsbruck, Department of General, Inorganic and Theoretical Chemistry, University of Innsbruck, Innsbruck, Austria
[3]Department of Integrative Structural and Computational Biology, The Scripps Research Institute, La Jolla, CA 92037, USA
[4]Instituto Clodomiro Picado, Facultad de Microbiologia, Universidad de Costa Rica, San Jose, Costa Rica
[5]BioInnovation Institute, Copenhagen, Denmark

Corresponding authors:
Esperanza Rivera de Torre
erdto@dtu.dk

Jens Preben Morth
premo@dtu.dk

Andreas H. Laustsen
ahola@bio.dtu.dk

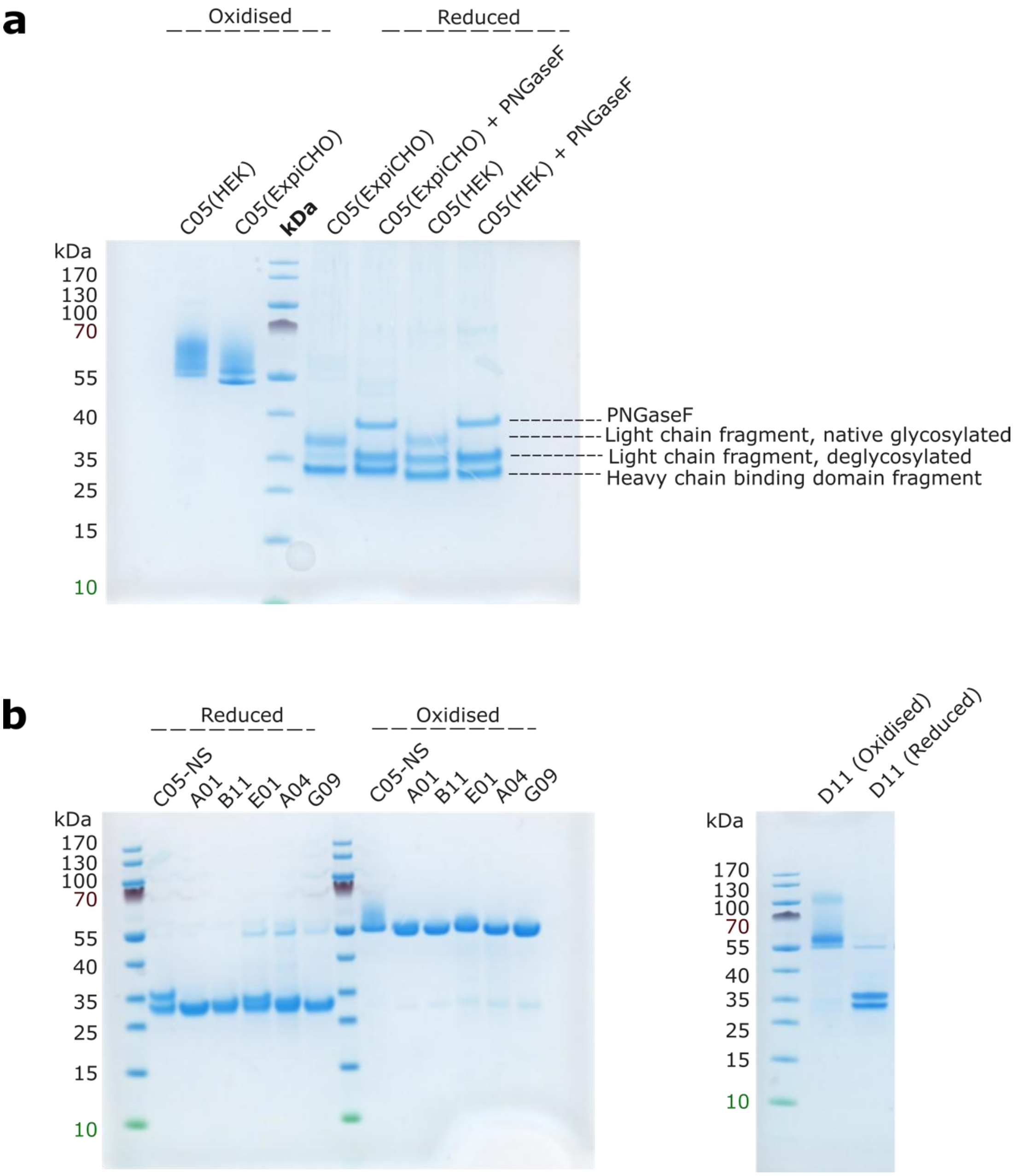

Supplementary Figure S1. SDS-PAGE gel analysis of Fab fragments for BLI binding assays. a. Analysis of the parental C05 Fab produced under reducing and non-reducing conditions with and without PNGase F treatment. The gel shows a clear reduction in molecular weight for the light chain following incubation with PNGase F, indicating that the N-linked glycosylation site (underlined) in the CDR-L3 loop (QSYDSSNGSVV) is exposed and glycosylated when produced in mammalian cells. **b.** SDS-PAGE analysis of the C05 mAb and chain-shuffled mAbs produced in ExpiCHO cells. The gels show the N-glycosylation site is removed in the C05 mAb by substituting Asn95a^{CDR-L3} with Ser (C05-NS).

Alt. text: *Two-panel figure showing SDS-PAGE analysis of Fab fragments used in biolayer interferometry (BLI) assays. Panel a (top) displays a Coomassie-stained gel comparing the parental C05 Fab under reducing and non-reducing conditions, with and without PNGase F treatment. A shift in molecular weight of the light chain after PNGase F treatment indicates N-linked glycosylation at the*

CDR-L3 loop. Panel b (bottom) shows SDS-PAGE of the full C05 mAb and chain-shuffled mAbs produced in ExpiCHO cells, demonstrating removal of the glycosylation site in the C05-NS variant via substitution of Asn95a with Ser.

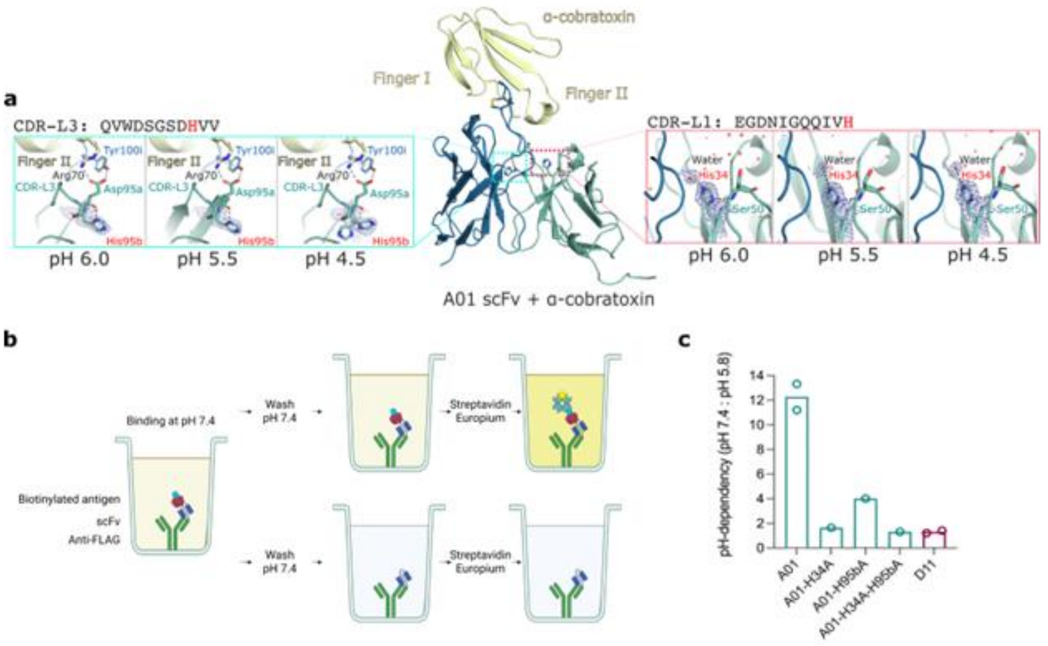

Supplementary Figure S2. Mutating histidine residues in the A01 light chain reduces pH-dependent binding of A01 to α-cobratoxin. a. A01 single-chain variable fragment (scFv) crystal structures at pH 6.0 (PDB: 9HXO), pH 5.5 (PDB: 9HUO), and pH 4.5 (PDB: 9FYT) showing hydrogen bond networks of histidine residues in the CDR-L1 and CDR-L3 loops. 2Fo-Fc density maps show His95b$^{CDR\text{-}L3}$ side-chain conformational changes, whereas no conformational changes are observed for His34$^{CDR\text{-}L1}$. **b.** DELFIA assay schematic to screen for pH-dependent binding of scFvs. **c.** The pH-dependent binding of the original A01 scFv with histidine residues substituted to alanine residues and the original D11 scFv.

Alt. text: *Three-panel figure demonstrating the impact of histidine mutations in the A01 light chain on pH-dependent binding to α-cobratoxin. Panel a (top) shows close-up views of crystal structures of A01 scFv bound to α-cobratoxin at pH 6.0, 5.5, and 4.5, highlighting hydrogen bonding networks involving histidine residues in the CDR-L1 and CDR-L3 loops. Electron density maps reveal a conformational change in the side chain of His95b in CDR-L3 across pH conditions, while His34 in CDR-L1 remains unchanged. Panel b (bottom-left) is a schematic diagram of the DELFIA assay used to evaluate pH-dependent binding. Panel c (bottom-right) presents a bar graph comparing binding of wild-type A01 scFv, histidine-to-alanine variants, and D11 scFv at different pH values.*

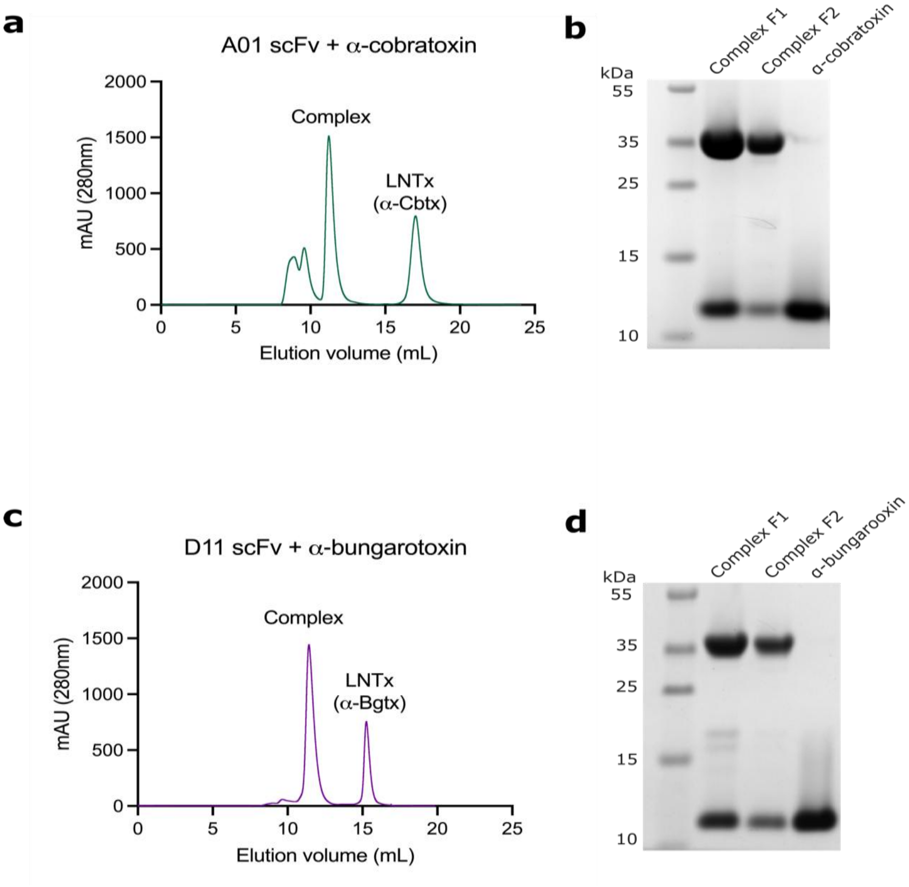

Supplementary Figure S3. Purification of A01 and D11 scFvs bound to LNTx for crystallographic studies. a, Size-exclusion chromatography profiles and **b**, SDS-PAGE analysis of the A01 scFv preincubated with α-cobratoxin (α-Cbtx); **c,d.** the same for the D11 scFv preincubated with α-bungarotoxin (α-Bgtx). The SEC fraction Complex Fraction 1 (Complex F1) identifies both scFv and LNTx and was selected for crystallization.

Alt. text: *Four-panel figure showing purification of A01 and D11 scFvs bound to long-chain α-neurotoxins (LNTxs) for crystallographic analysis. Top panels (a and b) display the size-exclusion chromatography (SEC) profile and corresponding SDS-PAGE analysis of A01 scFv preincubated with α-cobratoxin. Bottom panels (c and d) show the same for D11 scFv preincubated with α-bungarotoxin. In both cases, the SEC peak labeled "Complex Fraction 1 (Complex F1)" contains both scFv and toxin, confirming complex formation and suitability for crystallization. SDS-PAGE gels confirm the presence of both components in the selected fraction.*

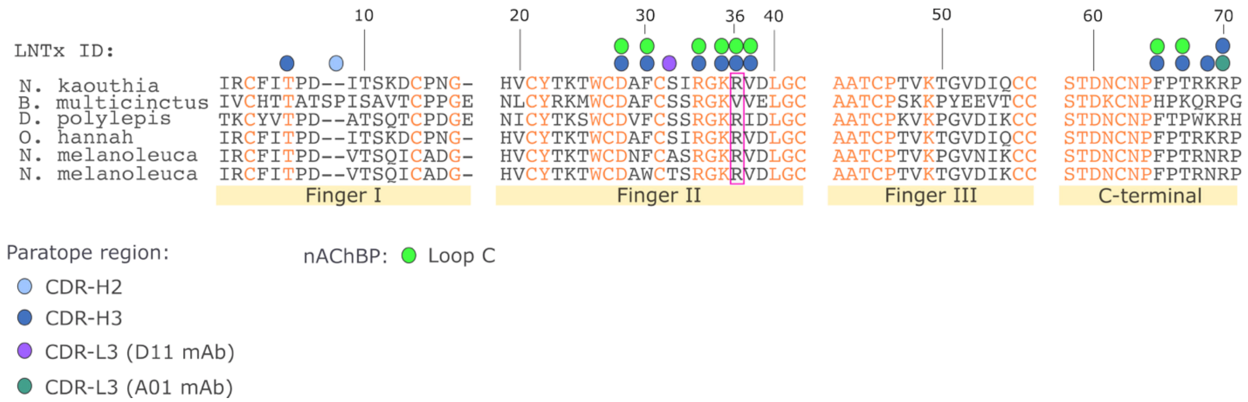

Supplementary Figure S4. Epitopes of the D11 and A01 mAbs to LNTx. Annotated epitope alignment of LNTxs with known reactivity with the D11 chain-shuffled mAb: *B. multicinctus* (α-bungarotoxin) (GenBank: AY678215, UniProt accession: P60615); *N. kaouthia* (α-cobratoxin) (GenBank: M94191, UniProt accession: P01391); *D. polylepis* (α-elapitoxin) (Genbank: M11373, Uniprot accession: P01396; *O. hannah* (Genbank: AY780085, Uniprot accession: A8N285); *N. melanoleuca* (Genbank: M11214, Uniprot accession: P01388 (top) and P0DQQ2 (bottom)). Residues conserved between LNTxs are coloured orange. The positions on LNTxs that contact the D11 and A01 mAbs and overlap with Loop C in nAChR are shown in circles above the primary sequence of LNTxs. Antibody-LNTx contacts were identified using PISA software and verified in PyMOL. Circles are colour coded by their respective loops. The critical epitope residue at position 36 in Finger II—arginine in *N. kaouthia* LNTx and valine in *B. multicinctus*—is highlighted in purple.

Alt. text: *Sequence alignment of long-chain α-neurotoxins from multiple snake species, annotated with known epitope residues for the D11 and A01 monoclonal antibodies. Conserved residues across the LNTxs are highlighted in orange. Colored circles above the sequences indicate contact points with D11 and A01, as determined by structural analysis (PISA and PyMOL), and correspond to specific structural loops, including Loop C of the nicotinic acetylcholine receptor (nAChR). A key epitope residue at position 36 in Finger II—arginine in N. kaouthia and valine in B. multicinctus—is emphasized in purple, reflecting its role in modulating antibody binding.*

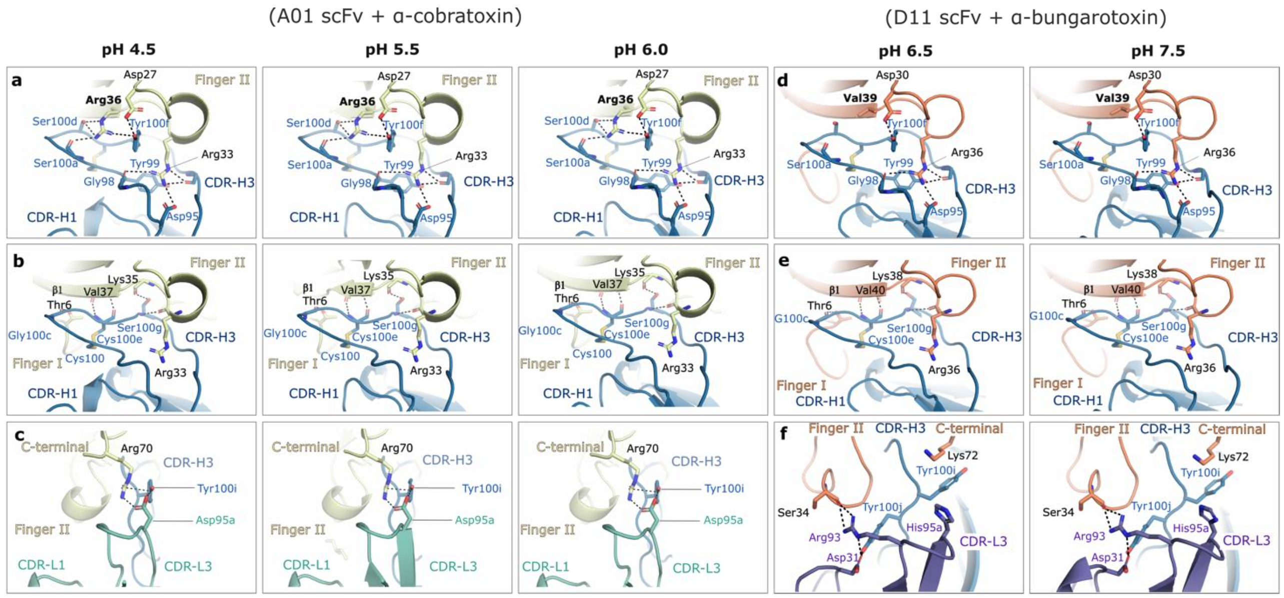

Supplementary Figure S5. Polar interactions between the A01 and D11 scFvs with LNTxs at different pHs. **a.** Interactions between the A01 V_H domain (blue) and the side-chain residues of α-cobratoxin (pale yellow) **b**. Backbone residues of α-cobratoxin at different pHs. **c**. Interactions between the A01 scFv and the C-terminal of α-cobratoxin. **d-f**. The same perspectives but for the D11 scFv interactions with α-bungarotoxin. Interactions between the V_H domain of both antibodies to the different LNTxs are equivalent at different pHs, except for the reduced hydrogen bonds at position Arg36 (Val39 on α-bungarotoxin). Interactions with the C-terminal tail are also reduced for D11 with α-bungarotoxin, due to differences in the structure of the C-terminal tail. The polar interactions between the D11 V_L (purple) and A01 V_L (green) are equivalent at the different pHs tested.

Alt. text: *Six-panel figure showing structural close-ups of polar interactions between A01 and D11 scFvs with their respective α-neurotoxin targets at different pH levels. Panels a-c display interactions between A01 scFv and α-cobratoxin at pH 4.5, 5.5, and 6.0, including side-chain and backbone contacts and engagement with the C-terminal region. Panels d-f show equivalent views for D11 scFv bound to α-bungarotoxin at pH 6.5 and 7.5.*

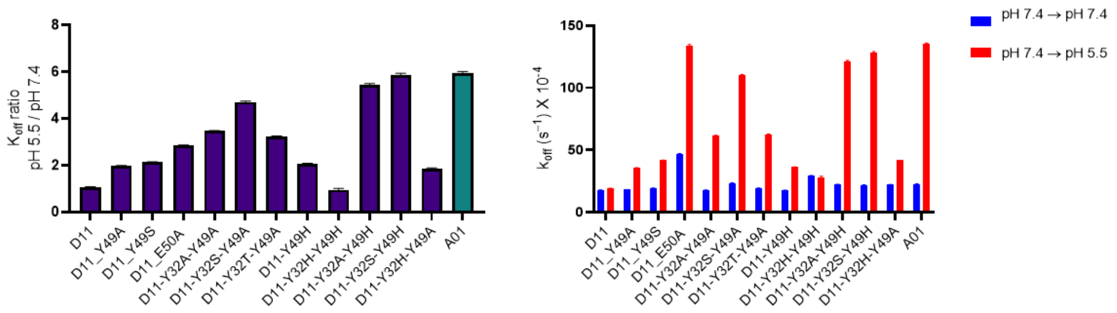

Supplementary Figure S6. **Biolayer interferometry (BLI) of A01, D11, and D11 mutants.** k_{off} ratio at pH 5.5 / pH 7.4 of D11 light chain mutant variants and the original D11 (purple) and A01 scFvs (teal) to α-cobratoxin. **b.** k_{off} values at pH 7.4 (blue) and pH 5.5 (red) determined in biolayer interferometry.

Alt. text: *Two-panel figure presenting biolayer interferometry (BLI) analysis of A01, D11, and D11 light-chain mutant scFvs binding to α-cobratoxin. The left panel shows a bar graph of the dissociation rate constant (koff) ratio at pH 5.5 vs. pH 7.4, highlighting pH sensitivity of binding. The right panel displays* k_{off} *and* k_{on} *values: association was measured at pH 7.4 (blue) and dissociation at pH 5.5 (red), for A01, D11, and D11 mutant variants. A01 values are shown in teal, and D11 in purple.*

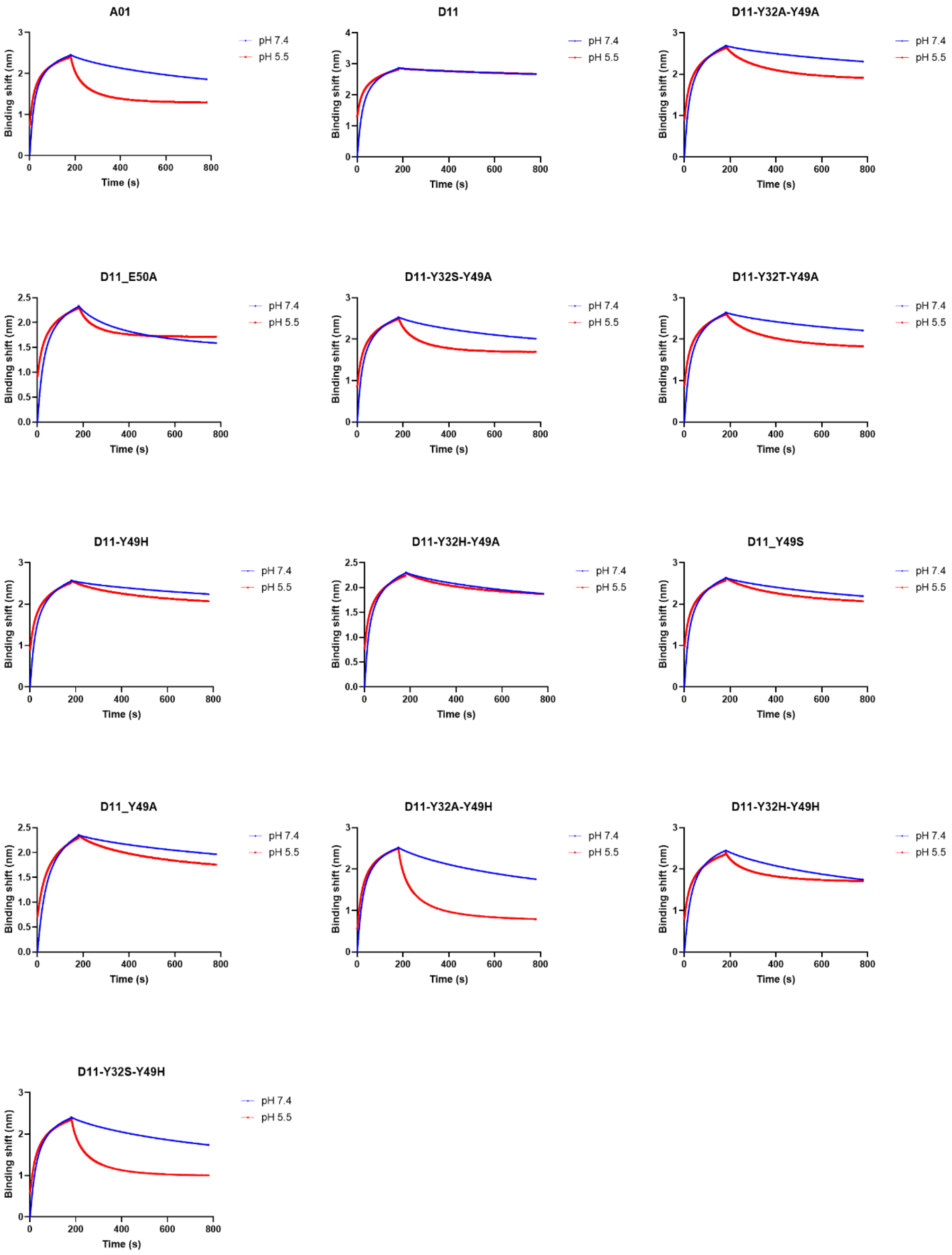

Supplementary Figure S7. Off-rate screening of D11 light-chain variant scFvs for pH-dependent binding to α-cobratoxin. BLI association and dissociation curves of scFvs binding to immobilised antigens.

Alt. text: *Biolayer interferometry (BLI) sensorgrams of D11 light-chain variant scFvs binding to immobilized α-cobratoxin. Curves represent association and dissociation phases used to screen for pH-dependent binding behavior. Variants were assessed for differences in off-rates under conditions mimicking physiological and endosomal pH.*

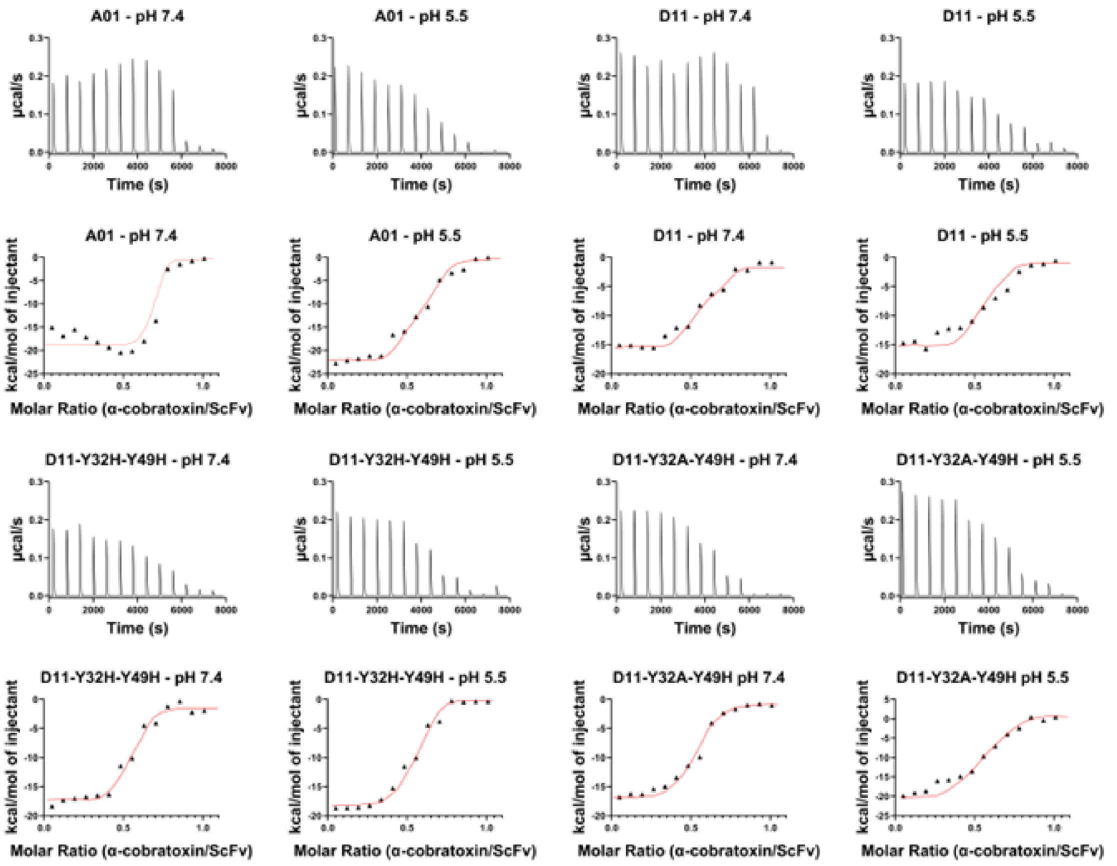

Sample	ΔH (kcal/mol)	n	Kd (nM)	ΔG (kcal/mol)	ΔS (kcal/mol·K)
**A01 - 7.4**	13.07 ± 0.12	0.8 ± 0.2	46.4 ± 7.3	-10.1 ± 2.3	0.08 ± 0.01
**A01 - 5.5**	13.20 ±0.08	0.7 ± 0.2	190.3 ± 4.2	-9.2 ± 2.1	0.08 ± 0.03
**D11 - 7.4**	9.51 ± 0.31	0.6 ± 0.2	31.3 ± 2.9	-10.2 ± 2.1	0.06 ± 0.01
**D11 - 5.5**	8.87 ± 0.21	0.6 ± 0.3	19.2 ± 1.3	-10.5 ± 0.9	0.06 ± 0.02
**D11-Y32H-Y49H - 7.4**	10.12 ± 0.82	0.6 ± 0.2	23.2 ± 3.8	-10.4 ± 2.5	0.07 ± 0.01
**D11-Y32H-Y49H - 5.5**	9.80 ± 0.34	0.6 ± 0.2	20.3 ± 4.5	-10.5 ± 2.3	0.07 ± 0.02
**D11-Y32A-Y49H - 7.4**	9.31±0.47	0.7 ± 0.3	31.6 ± 7.5	-10.2 ± 1.8	0.07 ± 0.02
**D11-Y32A-Y49H - 5.5**	10.47 ± 0.52	0.7 ± 0.4	119.2 ± 4.2	-9.4 ± 2.1	0.06 ± 0.02

Supplementary Figure S8. Isothermal titration calorimetry for A01, D11, D11-Y32H-Y49H, D11-Y32A-Y49H at pH 7.4 and 5.5. ITC data is fitted to a one-site model obtaining ΔH, n, and Kd. ΔG and ΔS are calculated based on these values and considering the temperature of the experiment run was 25 °C. Fitting curves are represented in red.

Alt. text: *Multi-panel figure displaying isothermal titration calorimetry (ITC) data for A01, D11, and two D11 light-chain variants (D11-Y32H-Y49H and D11-Y32A-Y49H) at pH 7.4 and pH 5.5. Each panel shows raw heat change traces and integrated binding curves fitted to a one-site binding model (red lines). Thermodynamic parameters including ΔH, binding stoichiometry (n), and Kd were derived from the fits; ΔG and ΔS were calculated assuming a temperature of 25 °C.*

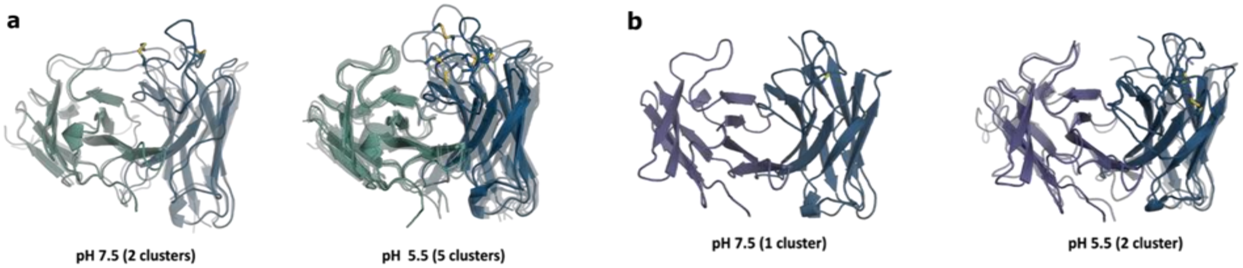

Supplementary Figure S9. pH-dependent conformational ensembles of A01 and D11 monoclonal antibodies to LNTx. a. A01 exhibits greater structural heterogeneity at pH 5.5 compared to D11. The heavy and light chains of A01 are shown in dark blue and green, respectively**. b.** D11 adopts a more restricted ensemble under identical conditions, with light and heavy chains colored purple and dark blue. The disulfide bond within the CDR-H3 loop is highlighted in yellow in both structures.

Alt. text: *Two-panel figure showing pH-dependent conformational ensembles of A01 and D11 monoclonal antibodies bound to long-chain α-neurotoxins (LNTx) at pH 5.5. Panel a illustrates the A01 scFv with heavy and light chains colored dark blue and green, respectively, displaying greater structural heterogeneity. Panel b shows the D11 scFv, with heavy and light chains in dark blue and purple, respectively, adopting a more restricted ensemble. In both structures, the disulfide bond within the CDR-H3 loop is highlighted in yellow.*

Supplementary Table S1. Binding kinetics between the anti-LNTx Fabs and α-cobratoxin BLI association and dissociation rates of Fabs binding to immobilised antigens were fitted using a 1:1 global binding model. Data represent three experimental repeats.

mAb	K_D (nM)	k_a ($M^{-1}s^{-1}$) (10^4)	$k_{dis\ pH\ 7.4}$ (s^{-1}) (10^{-4})	$k_{dis\ pH\ 5.5}$ (s^{-1}) (10^{-4})	pH 5.5 /pH 7.4
**A01**	21.0 ± 11.1	4.4 ± 3.0	7.5 ± 3.7	146.0 ± 6.1	19.7 ± 9.9
**G09**	4.2 ± 2.8	7.1 ± 5.4	2.1 ± 0.4	11.6 ± 1.7	5.6 ± 0.1
**A04**	15.3 ± 14.0	6.2 ± 4.0	5.9 ± 0.8	38.8 ± 12.0	6.6 ± 1.9
**B11**	5.7 ± 1.1	5.9 ± 0.8	3.4 ± 1.1	17.9 ± 1.7	5.6 ± 1.4
**C05**	71.2 ± 5.4	5.1 ± 0.8	36.6 ± 0.9	94.6 ± 1.1	2.6 ± 0.3
**A12**	4.2 ± 0.9	5.5 ± 2.1	2.1 ± 0.5	5.3 ± 0.2	2.5 ± 0.0
**D11**	1.8 ± 0.5	6.5 ± 1.3	1.2 ± 0.1	2.9 ± 0.5	2.5 ± 0.2
**E01**	6.6 ± 0.9	5.4 ± 1.5	3.5 ± 0.6	11.5 ± 1.3	3.4 ± 0.2

Supplementary Table S2. Binding kinetics between the anti-LNTx Fabs and α-elapitoxin BLI association and dissociation rates of Fabs binding to immobilised antigens were fitted using a 1:1 global binding model. Data represent three experimental repeats.

mAb	K_D (nM)	k_a ($M^{-1}s^{-1}$) (10^4)	$k_{dis\ pH\ 7.4}$ (s^{-1}) (10^{-4})	$k_{dis\ pH\ 5.5}$ (s^{-1}) (10^{-4})	pH 5.5 /pH 7.4
**A01**	2.1 ± 0.1	8.4 ± 2.1	1.7 ± 0.6	29.5 ± 4.3	16.9 ± 2.8
**G09**	1.0 ± 0.5	15.6 ± 6.1	1.5 ± 0.3	3.5 ± 0.4	2.4 ± 0.2
**A04**	0.6 ± 0.4	16.1 ± 9.0	0.9 ± 0.4	2.4 ± 1.1	2.5 ± 2.2
**B11**	1.4 ± 0.1	6.9 ± 0.8	0.9 ± 0.0	4.9 ± 1.5	5.2 ± 1.8
**C05**	4.6 ± 0.47	7.6 ± 1.63	3.4 ± 0.4	7.2 ± 2.2	2.1 ± 0.6
**A12**	1.4 ± 1.2	14.0 ± 7.7	1.5 ± 0.7	3.6 ± 1.0	2.4 ± 1.1
**D11**	1.4 ± 0.6	12.1 ± 5.2	1.5 ± 0.3	4.1 ± 1.3	2.8 ± 0.5
**E01**	1.3 ± 0.7	13.8 ± 7.9	1.4 ± 0.3	4.9 ± 0.7	3.5 ± 0.4

Supplementary Table S3. Unfolding temperatures (T_m) of anti-LNTx Fab fragments at pH 7.4 and pH 5.5 The thermal stability of the pH-dependent A01 Fab at pH 7.4 and pH 5.5 compared with representative pH-independent mAb Fabs with an IGVL3-21 light chain (G09) or an IGVL6-57 light chain (D11) and the parental Fab (C05).

mAb	pH	Average T_m (ºC)
**A01**	7.4	74.30 ± 0.03
**A01**	5.5	73.95 ± 0.02
**G09**	7.4	72.79 ± 0.01
**G09**	5.5	73.13 ± 0.07
**D11**	7.4	77.85 ± 0.15
**D11**	5.5	78.61 ± 0.04
**C05**	7.4	75.73 ± 0.51
**C05**	5.5	75.96 ± 0.01

Supplementary Table S4. Data collection and refinement statistics of A01 and α-cobratoxin and D11 and α-bungarotoxin scFv-LNTx complexes.

	D11 α-Bgtx pH 6.5	D11 α-Bgtx pH 7.5	A01 α-Cbtx pH 4.5	A01 α-Cbtx pH 5.5	A01 α-Cbtx pH 6.0
**PDB ID**	9FYS	9HUB	9FYT	9HUO	9HXO
**Wavelength (Å)**	1.033	1.033	0.9763	0.9537	0.9537
**Resolution range (Å)**	21.12-1.32 (1.36-1.32)	22.38-1.33 (1.34-1.33)	42.52-1.55 (1.605-1.55)	41.58-1.6 (1.62-1.6)	60.57-1.49 (1.52-1.49)
**Space group**	P 21 21 21	P 21 21 21	P 21 21 21	P 21 21 21	P 21 21 21
**Unit cell at 90° (Å)**	79.84, 83.80, 102.78	79.05, 84.73, 102.82	76.86, 83.86, 98.65	76.87, 83.15, 98.34	76.94, 82.65, 98.19
**Total reflections**	2132700 (176681)	2108521 (55898)	486522 (47473)	419481 (8796)	
**Unique reflections**	158875 (14966)	158613 (5216)	92830 (9112)	83281 (2739)	53423 (415)
**Multiplicity**	13.4 (11.8)	13.3 (10.7)	5.2 (5.2)	5.0 (3.2)	13.3 (12.9)
**Completeness (%)**	97.20 (84.1)	99.61 (91.6)	99.64 (98.8)	99.37 (97.5)	52.03 (7.8)
**Mean I/sigma(I)**	8.99 (0.36)	8.44 (0.31)	10.86 (1.20)	10.0 (1.0)	10.3 (1.6)
**Wilson B-factor**	18.5	18.9	21.4	20.7	14.0
**R-merge**	0.1114 (>1)	0.1228 (3.716)	0.07046 (>1)	0.0672 (0.888)	0.171 (>1)
**R-meas**	0.116 (>1)	0.127 (3.90)	0.0783 (>1)	0.075 (>1)	0.178 (>1)
**R-pim**	0.031 (0.48)	0.034 (1.17)	0.033 (0.69)	0.033 (0.54)	0.048 (0.48)
**CC1/2**	0.98 (0.47)	0.99 (0.34)	0.99 (0.69)	0.99 (0.71)	0.99 (0.63)
**CC***	0.99 (0.80)	0.99 (0.71)	1.00 (0.90)	1.00 (0.91)	1.00 (0.89)
**Reflections used in refinement**	157301 (13476)	158001 (4812)	92665 (9087)	83128 (2736)	53423 (415)
**Reflections used for R-free**	7968 (723)	8021 (270)	4598 (466)	4075 (151)	2612 (23)
**R-work**	0.169 (0.409)	0.168 (0.432)	0.198 (0.419)	0.183 (0.412)	0.204 (0.300)
**R-free**	0.168 (0.401)	0.197 (0.428)	0.225 (0.436)	0.225 (0.423)	0.248 (0.242)
**Number of non-hydrogen atoms**	5927	6128	5477	5504	5601
**Macromolecules**	5038	5131	4913	4913	4925
**Ligands**	26	28	78	39	39
**Solvent**	876	969	523	552	637
**Protein residues**	651	652	634	634	634
**RMS (bonds)**	0.009	0.008	0.005	0.007	0.004
**RMS (angles)**	1.09	0.99	0.75	0.92	0.67
**Ramachandran favored (%)**	97.81	97.19	96.46	96.14	95.66
**Ramachandran allowed (%)**	2.19	2.81	3.38	3.54	4.18
**Ramachandran outliers (%)**	0.00	0.00	0.16	0.32	0.16
**Rotamer outliers (%)**	0.70	0.69	1.29	1.29	1.1
**Clashscore**	2.14	2.09	3.10	3.53	2.08
**Average B-factor**	26.01	27.8	34.29	30.8	21.8

**Macromolecules**	24.32	25.3	33.71	30.2	21.3
**Ligands**	56.24	44.67	51.01	46.7	32.3
**Solvent**	35.24	40.3	38.35	34.9	24.3
**Number of TLS groups**	33	33	35	35	35

*Statistics for the highest-resolution shell are shown in parentheses.

Supplementary Table S5. BLI association and dissociation rates of the D11 Fab bound to α-bungarotoxin fit using a 1:1 global binding model. Data represent three experimental repeats.

mAb	K_D (nM)	k_a ($M^{-1}s^{-1}$) ($\times 10^4$)	$k_{dis\ pH\ 7.4}$ (s^{-1}) ($\times 10^{-3}$)	$k_{dis\ pH\ 5.5}$ (s^{-1}) ($\times 10^{-3}$)	pH 5.5 /pH 7.4
**D11**	115.9 ± 19.6	1.3 ± 0.3	4.7 ± 0.3	5.0 ± 0.8	1.1 ± 0.4

Supplementary Table S6. BLI affinity measurements of Fab fragments bound to α-bungarotoxin Affinities were measured either by affinity kinetics or under steady-state conditions. Curves were fitted using either a 1:1 global binding model when determined by affinity kinetics or a one-site-specific binding model. Data represent three experimental repeats.

mAb	Method	Model	K_D (nM)	k_a ($M^{-1}s^{-1}$) ($\times 10^4$)	$k_{dis\ pH\ 7.4}$ (s^{-1}) ($\times 10^{-3}$)
**A01**	Steady state	One-site-specific	3,090 ± 164	N/A	N/A
**G09**	Steady state	One-site-specific	3,160 ± 779	N/A	N/A
**A04**	Steady state	One-site-specific	963.0 ± 380	N/A	N/A
**B11**	Steady state	One-site-specific	3,390 ± 202	N/A	N/A
**C05**	Steady state	One-site-specific	3,930 ± 864	N/A	N/A
**A12**	Affinity kinetics	1:1 Global fit	202.0 ± 28.2	2.1 ± 0.5	9.3 ± 0.8
**D11**	Affinity kinetics	1:1 Global fit	115.9 ± 19.6	1.3 ± 0.3	4.7 ± 0.3
**E01**	Affinity kinetics	1:1 Global fit	270.6 ± 31.1	2.3 ± 0.5	17.3 ± 0.04

Supplementary Table S7. Interaction energies between water and His34$^{CDR\text{-}L1}$ from molecular dynamics simulations at pH 7.5 and pH 5.5.

mAb	pH7.5/kcal/mol	pH5.5/kcal/mol
**A01**	-9 ± 3	-52 ± 15
**D11**	-8 ± 4	-7 ± 4
**D11-Y32S-Y49A**	-8 ± 4	-50 ± 18
**D11-Y32H-Y49A**	-8 ± 10	-44 ± 32

Supplementary Table S8. Binding kinetics between the D11 light chain mutant variants and the original D11 and A01 scFvs and α-cobratoxin BLI association and dissociation rates of scFvs binding to immobilised antigens were fitted using a 1:1 global binding model.

scFv	K_D (nM)	k_a ($M^{-1}s^{-1}$) ($\times10^4$)	$k_{dis\ pH\ 7.4}$ (s^{-1}) ($\times10^{-4}$)	$k_{dis\ pH\ 5.5}$ (s^{-1}) ($\times10^{-4}$)	pH 5.5 /pH 7.4
**A01**	52.20	4.32	22.55 ± 0.15	134.5 ± 1.1	5.96 ± 0.06
**D11**	44.41	3.99	17.73 ± 0.21	18.87 ± 0.23	1.06 ± 0.02
**D11_E50A**	157.90	2.97	46.92 ± 0.28	133.7 ± 0.78	2.85 ± 0.02
**D11-Y32A-Y49A**	45.36	3.92	17.76 ± 0.14	61.39 ± 0.36	3.46 ± 0.03
**D11-Y32S-Y49A**	60.86	3.84	23.4 ± 0.15	110.3 ± 0.71	4.71 ± 0.04
**D11-Y32T-Y49A**	52.07	3.71	19.32 ± 0.14	62.37 ± 0.39	3.23 ± 0.03
**D11-Y49H**	47.84	3.67	17.53 ± 0.16	36.14 ± 0.22	2.06 ± 0.02
**D11-Y32H-Y49A**	56.06	3.97	22.23 ± 0.14	41.45 ± 0.22	1.86 ± 0.02
**D11_Y49S**	48.82	4.00	19.5 ± 0.12	41.62 ± 0.21	2.13 ± 0.02
**D11_Y49A**	68.77	2.62	18.04 ± 0.13	35.49 ± 0.22	1.96 ± 0.02
**D11-Y32A-Y49H**	60.80	3.67	22.3 ± 0.13	121.4 ± 1.12	5.44 ± 0.06
**D11-Y32H-Y49H**	88.4	3.33	29.3 ± 0.12	27.7 ± 1.06	0.95 ± 0.06
**D11-Y32S-Y49H**	64.16	3.40	21.8 ± 0.13	128.1 ± 1.03	5.88 ± 0.06
