# Supplementary material for: Rational design of antibodies with pH-dependent antigen-binding properties using structural insights from broadly neutralizing antibodies against α-neurotoxins
Source: MAbs. 2025 Sep 11;17(1):2553624. doi: 10.1080/19420862.2025.2553624 (PMC12439581; doi:10.1080/19420862.2025.2553624)
Supplement: D1292139980valreportfullP1.pdf [file KMAB_A_2553624_SM1038.pdf]

Full wwPDB X-ray Structure Validation Report (i)

Jul 4, 2024 – 09:54 am BST

PDB ID	:	9FYS
Title	:	D11 mAbs bound to alpha-Bungarotoxin
Deposited on	:	2024-07-03
Resolution	:	1.32 Å(reported)

This wwPDB validation report is for manuscript review

This is a Full wwPDB X-ray Structure Validation Report.

This report is produced by the wwPDB biocuration pipeline after annotation of the structure.

We welcome your comments at *validation@mail.wwpdb.org*
A user guide is available at
https://www.wwpdb.org/validation/2017/XrayValidationReportHelp
with specific help available everywhere you see the (i) symbol.

The types of validation reports are described at
http://www.wwpdb.org/validation/2017/FAQs#types.

The following versions of software and data (see references (i)) were used in the production of this report:

MolProbity	:	4.02b-467
Mogul	:	1.8.4, CSD as541be (2020)
Xtriage (Phenix)	:	1.13
EDS	:	2.37.1
Percentile statistics	:	20191225.v01 (using entries in the PDB archive December 25th 2019)
Refmac	:	5.8.0158
CCP4	:	7.0.044 (Gargrove)
Ideal geometry (proteins)	:	Engh & Huber (2001)
Ideal geometry (DNA, RNA)	:	Parkinson et al. (1996)
Validation Pipeline (wwPDB-VP)	:	2.37.1

For Manuscript Review

1 Overall quality at a glance (i)

The following experimental techniques were used to determine the structure:
X-RAY DIFFRACTION

The reported resolution of this entry is 1.32 Å.

Percentile scores (ranging between 0-100) for global validation metrics of the entry are shown in the following graphic. The table shows the number of entries on which the scores are based.

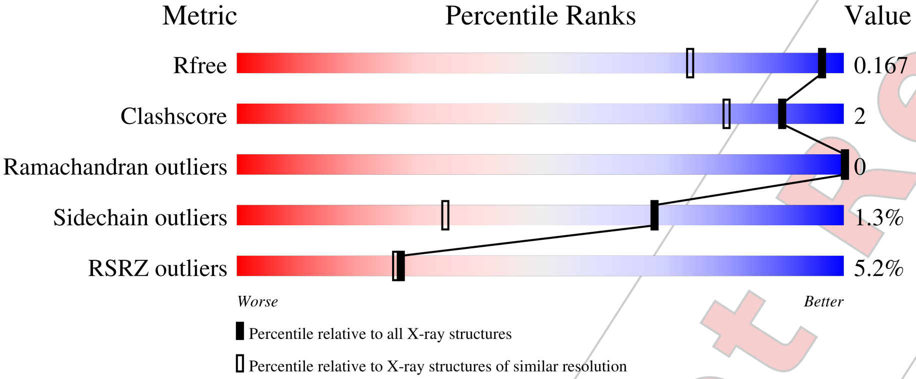

Metric	Whole archive (#Entries)	Similar resolution (#Entries, resolution range(Å))
R_{free}	130704	1611 (1.34-1.30)
Clashscore	141614	1667 (1.34-1.30)
Ramachandran outliers	138981	1615 (1.34-1.30)
Sidechain outliers	138945	1615 (1.34-1.30)
RSRZ outliers	127900	1580 (1.34-1.30)

The table below summarises the geometric issues observed across the polymeric chains and their fit to the electron density. The red, orange, yellow and green segments of the lower bar indicate the fraction of residues that contain outliers for >=3, 2, 1 and 0 types of geometric quality criteria respectively. A grey segment represents the fraction of residues that are not modelled. The numeric value for each fraction is indicated below the corresponding segment, with a dot representing fractions <=5% The upper red bar (where present) indicates the fraction of residues that have poor fit to the electron density. The numeric value is given above the bar.

Mol	Chain	Length	Quality of chain
1	C	121	7% / 96% • •
1	M	121	5% / 93% 5% • •
2	X	95	4% / 67% 7% • 24%
2	Z	95	2% / 73% 5% 22%

Continued on next page...

For Manuscript Review

Continued from previous page...

Mol	Chain	Length	Quality of chain
3	B	144	4% 88% • • 8%
3	I	144	6% 83% 9% 8%

For Manuscript Review

2 Entry composition (i)

There are 5 unique types of molecules in this entry. The entry contains 10693 atoms, of which 4767 are hydrogens and 0 are deuteriums.

In the tables below, the ZeroOcc column contains the number of atoms modelled with zero occupancy, the AltConf column contains the number of residues with at least one atom in alternate conformation and the Trace column contains the number of residues modelled with at most 2 atoms.

- Molecule 1 is a protein called heavy chain.

Mol	Chain	Residues	Atoms						ZeroOcc	AltConf	Trace
1	C	119	Total 1794	C 569	H 866	N 157	O 197	S 5	0	6	0
1	M	120	Total 1810	C 574	H 870	N 160	O 201	S 5	11	6	0

- Molecule 2 is a protein called Alpha-bungarotoxin.

Mol	Chain	Residues	Atoms						ZeroOcc	AltConf	Trace
2	X	72	Total 1067	C 333	H 525	N 95	O 103	S 11	0	1	0
2	Z	74	Total 1081	C 338	H 530	N 97	O 105	S 11	0	0	0

- Molecule 3 is a protein called D11 mAbs Light chain.

Mol	Chain	Residues	Atoms						ZeroOcc	AltConf	Trace
3	I	133	Total 1998	C 645	H 971	N 171	O 203	S 8	0	4	0
3	B	133	Total 2041	C 657	H 992	N 173	O 211	S 8	0	10	0

- Molecule 4 is 2-(N-MORPHOLINO)-ETHANESULFONIC ACID (three-letter code: MES) (formula: $C_6H_{13}NO_4S$).

For Manuscript Review

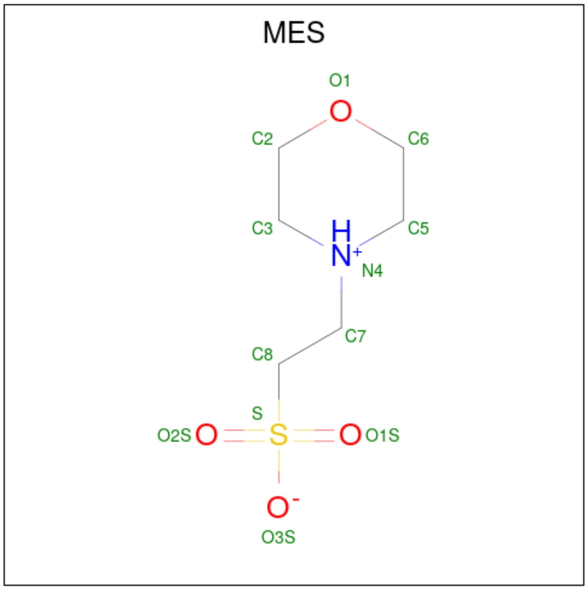

Mol	Chain	Residues	Atoms	ZeroOcc	AltConf
4	Z	1	Total C H N O S<br>25 6 13 1 4 1	0	0

- Molecule 5 is water.

Mol	Chain	Residues	Atoms	ZeroOcc	AltConf
5	C	147	Total O<br>147 147	0	0
5	X	93	Total O<br>93 93	0	0
5	Z	87	Total O<br>87 87	0	0
5	I	167	Total O<br>167 167	0	0
5	M	185	Total O<br>185 185	0	0
5	B	198	Total O<br>198 198	0	0

For Manuscript Review

3 Residue-property plots (i)

These plots are drawn for all protein, RNA, DNA and oligosaccharide chains in the entry. The first graphic for a chain summarises the proportions of the various outlier classes displayed in the second graphic. The second graphic shows the sequence view annotated by issues in geometry and electron density. Residues are color-coded according to the number of geometric quality criteria for which they contain at least one outlier: green = 0, yellow = 1, orange = 2 and red = 3 or more. A red dot above a residue indicates a poor fit to the electron density (RSRZ > 2). Stretches of 2 or more consecutive residues without any outlier are shown as a green connector. Residues present in the sample, but not in the model, are shown in grey.

• Molecule 1: heavy chain

Chain C:
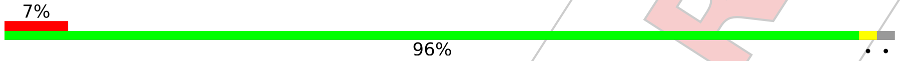

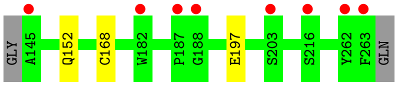

• Molecule 1: heavy chain

Chain M:
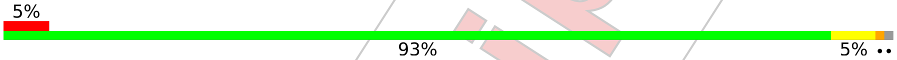

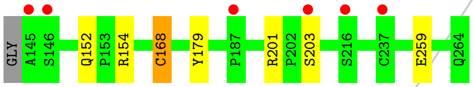

• Molecule 2: Alpha-bungarotoxin

Chain X:
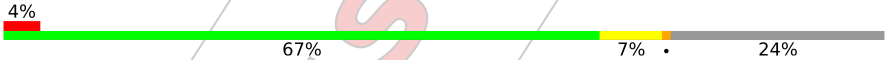

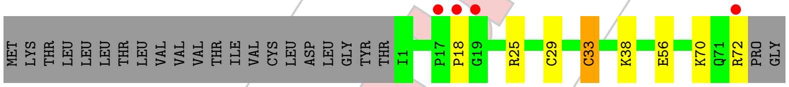

• Molecule 2: Alpha-bungarotoxin

Chain Z:
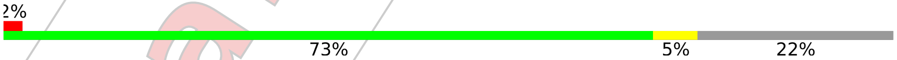

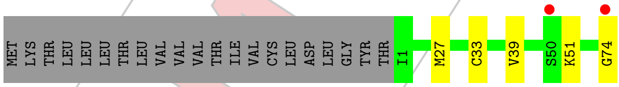

• Molecule 3: D11 mAbs Light chain

Chain I:
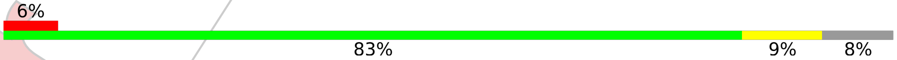

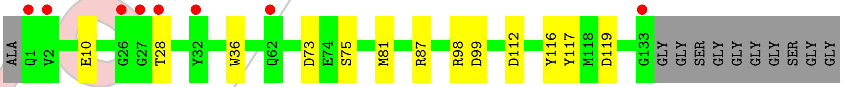

• Molecule 3: D11 mAbs Light chain

For Manuscript Review

Chain B:

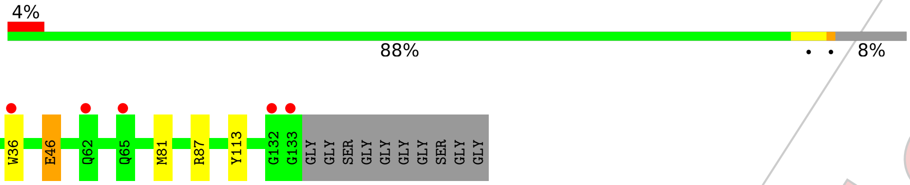

For Manuscript Review

4 Data and refinement statistics (i)

Property	Value	Source
Space group	P 21 21 21	Depositor
Cell constants<br>a, b, c, α, β, γ	79.85Å 83.80Å 102.78Å<br>90.00° 90.00° 90.00°	Depositor
Resolution (Å)	25.17 – 1.32<br>22.09 – 1.32	Depositor<br>EDS
% Data completeness<br>(in resolution range)	95.2 (25.17-1.32)<br>95.3 (22.09-1.32)	Depositor<br>EDS
R_{merge}	(Not available)	Depositor
R_{sym}	(Not available)	Depositor
$< I/\sigma(I) >$ [1]	0.97 (at 1.32Å)	Xtriage
Refinement program	PHENIX 1.20.1_4487	Depositor
R, R_{free}	0.167 , 0.192<br>0.168 , 0.167	Depositor<br>DCC
R_{free} test set	7820 reflections (5.06%)	wwPDB-VP
Wilson B-factor (Å^2)	18.6	Xtriage
Anisotropy	0.263	Xtriage
Bulk solvent k_{sol}(e/Å^3), B_{sol}(Å^2)	0.40 , 44.6	EDS
L-test for twinning[2]	$< \|L\| > = 0.49$, $< L^2 > = 0.32$	Xtriage
Estimated twinning fraction	0.020 for k,h,-l	Xtriage
F_o,F_c correlation	0.98	EDS
Total number of atoms	10693	wwPDB-VP
Average B, all atoms (Å^2)	28.0	wwPDB-VP

Xtriage's analysis on translational NCS is as follows: *The analyses of the Patterson function reveals a significant off-origin peak that is 28.44 % of the origin peak, indicating pseudo-translational symmetry. The chance of finding a peak of this or larger height randomly in a structure without pseudo-translational symmetry is equal to 1.8412e-03. The detected translational NCS is most likely also responsible for the elevated intensity ratio.*

[1] Intensities estimated from amplitudes.
[2] Theoretical values of $< |L| >$, $< L^2 >$ for acentric reflections are 0.5, 0.333 respectively for untwinned datasets, and 0.375, 0.2 for perfectly twinned datasets.

For Manuscript Review

5 Model quality (i)

5.1 Standard geometry (i)

Bond lengths and bond angles in the following residue types are not validated in this section: MES

The Z score for a bond length (or angle) is the number of standard deviations the observed value is removed from the expected value. A bond length (or angle) with $|Z| > 5$ is considered an outlier worth inspection. RMSZ is the root-mean-square of all Z scores of the bond lengths (or angles).

Mol	Chain	Bond lengths		Bond angles	
		RMSZ	#$\|Z\|$ >5	RMSZ	#$\|Z\|$ >5
1	C	0.85	1/973 (0.1%)	0.86	0/1321
1	M	0.93	2/984 (0.2%)	0.89	0/1334
2	X	0.91	0/558	0.97	2/758 (0.3%)
2	Z	0.90	0/565	0.96	0/767
3	B	0.97	3/1117 (0.3%)	0.99	0/1512
3	I	1.03	3/1067 (0.3%)	1.05	6/1444 (0.4%)
All	All	0.94	9/5264 (0.2%)	0.96	8/7136 (0.1%)

Chiral center outliers are detected by calculating the chiral volume of a chiral center and verifying if the center is modelled as a planar moiety or with the opposite hand.A planarity outlier is detected by checking planarity of atoms in a peptide group, atoms in a mainchain group or atoms of a sidechain that are expected to be planar.

Mol	Chain	#Chirality outliers	#Planarity outliers
3	I	0	1

All (9) bond length outliers are listed below:

Mol	Chain	Res	Type	Atoms	Z	Observed(Å)	Ideal(Å)
3	I	117	TYR	CE1-CZ	-9.60	1.26	1.38
1	M	259	GLU	CB-CG	-7.41	1.38	1.52
3	B	12	LYS	CE-NZ	6.41	1.65	1.49
3	I	10	GLU	CD-OE1	6.40	1.32	1.25
1	C	197	GLU	CB-CG	-5.89	1.41	1.52
3	B	46[A]	GLU	CD-OE2	-5.82	1.19	1.25
3	B	46[B]	GLU	CD-OE2	-5.82	1.19	1.25
3	I	117	TYR	CD1-CE1	5.17	1.47	1.39
1	M	179	TYR	CE2-CZ	-5.10	1.31	1.38

All (8) bond angle outliers are listed below:

For Manuscript Review

Mol	Chain	Res	Type	Atoms	Z	Observed(°)	Ideal(°)
3	I	119	ASP	CB-CG-OD1	8.47	125.92	118.30
3	I	117	TYR	CG-CD1-CE1	-5.72	116.72	121.30
3	I	99[A]	ASP	CB-CG-OD2	-5.54	113.31	118.30
3	I	99[B]	ASP	CB-CG-OD2	-5.54	113.31	118.30
2	X	25	ARG	NE-CZ-NH2	-5.36	117.62	120.30
3	I	117	TYR	CZ-CE2-CD2	-5.13	115.18	119.80
3	I	112	ASP	CB-CG-OD1	5.06	122.85	118.30
2	X	18	PRO	N-CD-CG	-5.05	95.62	103.20

There are no chirality outliers.

All (1) planarity outliers are listed below:

Mol	Chain	Res	Type	Group
3	I	87	ARG	Sidechain

5.2 Too-close contacts (i)

In the following table, the Non-H and H(model) columns list the number of non-hydrogen atoms and hydrogen atoms in the chain respectively. The H(added) column lists the number of hydrogen atoms added and optimized by MolProbity. The Clashes column lists the number of clashes within the asymmetric unit, whereas Symm-Clashes lists symmetry-related clashes.

Mol	Chain	Non-H	H(model)	H(added)	Clashes	Symm-Clashes
1	C	928	866	843	2	0
1	M	940	870	841	3	0
2	X	542	525	527	4	0
2	Z	551	530	532	2	0
3	B	1049	992	951	5	0
3	I	1027	971	966	3	0
4	Z	12	13	13	0	0
5	B	198	0	0	1	0
5	C	147	0	0	0	0
5	I	167	0	0	0	0
5	M	185	0	0	1	0
5	X	93	0	0	0	0
5	Z	87	0	0	1	0
All	All	5926	4767	4673	18	0

The all-atom clashscore is defined as the number of clashes found per 1000 atoms (including hydrogen atoms). The all-atom clashscore for this structure is 2.

All (18) close contacts within the same asymmetric unit are listed below, sorted by their clash magnitude.

For Manuscript Review

Atom-1	Atom-2	Interatomic distance (Å)	Clash overlap (Å)
1:C:152:GLN:HG2	1:C:168[B]:CYS:SG	2.09	0.92
2:Z:74:GLY:OXT	5:Z:201:HOH:O	1.99	0.78
3:I:28:THR:O	3:I:98[B]:ARG:NH2	2.37	0.57
1:C:152:GLN:CG	1:C:168[B]:CYS:SG	2.93	0.52
2:X:70:LYS:HD3	3:B:113:TYR:CZ	2.45	0.52
1:M:152:GLN:CG	1:M:168[A]:CYS:SG	2.99	0.50
2:X:38:LYS:NZ	2:X:56:GLU:OE2	2.44	0.50
1:M:152:GLN:HG2	1:M:168[A]:CYS:SG	2.52	0.49
3:I:36:TRP:CE2	3:I:81[A]:MET:HB2	2.50	0.47
3:B:15:GLY:HA2	3:B:87:ARG:NH2	2.30	0.46
3:B:15:GLY:HA2	3:B:87:ARG:HH21	1.85	0.42
2:X:72:ARG:HH11	2:X:72:ARG:HG3	1.84	0.42
2:Z:27:MET:HA	2:Z:39:VAL:O	2.19	0.42
3:B:36:TRP:CE2	3:B:81[A]:MET:HB2	2.55	0.42
2:X:29:CYS:HB3	2:X:33:CYS:HB2	1.92	0.41
1:M:201:ARG:NH1	5:M:311:HOH:O	2.53	0.41
3:B:46[A]:GLU:HG3	5:B:229:HOH:O	2.19	0.41
3:I:73:ASP:OD1	3:I:75:SER:OG	2.27	0.41

There are no symmetry-related clashes.

5.3 Torsion angles (i)

5.3.1 Protein backbone (i)

In the following table, the Percentiles column shows the percent Ramachandran outliers of the chain as a percentile score with respect to all X-ray entries followed by that with respect to entries of similar resolution.

The Analysed column shows the number of residues for which the backbone conformation was analysed, and the total number of residues.

Mol	Chain	Analysed	Favoured	Allowed	Outliers	Percentiles	
1	C	123/121 (102%)	119 (97%)	4 (3%)	0	100	100
1	M	124/121 (102%)	120 (97%)	4 (3%)	0	100	100
2	X	71/95 (75%)	69 (97%)	2 (3%)	0	100	100
2	Z	72/95 (76%)	72 (100%)	0	0	100	100
3	B	141/144 (98%)	137 (97%)	4 (3%)	0	100	100
3	I	135/144 (94%)	133 (98%)	2 (2%)	0	100	100
All	All	666/720 (92%)	650 (98%)	16 (2%)	0	100	100

For Manuscript Review

There are no Ramachandran outliers to report.

5.3.2 Protein sidechains (i)

In the following table, the Percentiles column shows the percent sidechain outliers of the chain as a percentile score with respect to all X-ray entries followed by that with respect to entries of similar resolution.

The Analysed column shows the number of residues for which the sidechain conformation was analysed, and the total number of residues.

Mol	Chain	Analysed	Rotameric	Outliers	Percentiles	
1	C	112/107 (105%)	112 (100%)	0	100	100
1	M	113/107 (106%)	109 (96%)	4 (4%)	36	5
2	X	65/85 (76%)	64 (98%)	1 (2%)	65	31
2	Z	65/85 (76%)	63 (97%)	2 (3%)	40	6
3	B	115/108 (106%)	115 (100%)	0	100	100
3	I	110/108 (102%)	109 (99%)	1 (1%)	78	51
All	All	580/600 (97%)	572 (99%)	8 (1%)	69	33

All (8) residues with a non-rotameric sidechain are listed below:

Mol	Chain	Res	Type
2	X	33	CYS
2	Z	33	CYS
2	Z	51	LYS
3	I	116	TYR
1	M	154	ARG
1	M	168[A]	CYS
1	M	168[B]	CYS
1	M	203	SER

Sometimes sidechains can be flipped to improve hydrogen bonding and reduce clashes. There are no such sidechains identified.

5.3.3 RNA (i)

There are no RNA molecules in this entry.

For Manuscript Review

5.4 Non-standard residues in protein, DNA, RNA chains (i)

There are no non-standard protein/DNA/RNA residues in this entry.

5.5 Carbohydrates (i)

There are no monosaccharides in this entry.

5.6 Ligand geometry (i)

1 ligand is modelled in this entry.

In the following table, the Counts columns list the number of bonds (or angles) for which Mogul statistics could be retrieved, the number of bonds (or angles) that are observed in the model and the number of bonds (or angles) that are defined in the Chemical Component Dictionary. The Link column lists molecule types, if any, to which the group is linked. The Z score for a bond length (or angle) is the number of standard deviations the observed value is removed from the expected value. A bond length (or angle) with $|Z| > 2$ is considered an outlier worth inspection. RMSZ is the root-mean-square of all Z scores of the bond lengths (or angles).

Mol	Type	Chain	Res	Link	Bond lengths			Bond angles		
					Counts	RMSZ	$\#\|Z\| > 2$	Counts	RMSZ	$\#\|Z\| > 2$
4	MES	Z	101	-	12,12,12	1.43	1 (8%)	14,16,16	1.75	3 (21%)

In the following table, the Chirals column lists the number of chiral outliers, the number of chiral centers analysed, the number of these observed in the model and the number defined in the Chemical Component Dictionary. Similar counts are reported in the Torsion and Rings columns. '-' means no outliers of that kind were identified.

Mol	Type	Chain	Res	Link	Chirals	Torsions	Rings
4	MES	Z	101	-	-	5/6/14/14	0/1/1/1

All (1) bond length outliers are listed below:

Mol	Chain	Res	Type	Atoms	Z	Observed(Å)	Ideal(Å)
4	Z	101	MES	C8-S	-4.55	1.71	1.77

All (3) bond angle outliers are listed below:

Mol	Chain	Res	Type	Atoms	Z	Observed(^{o})	Ideal(^{o})
4	Z	101	MES	O2S-S-C8	3.87	111.58	106.92
4	Z	101	MES	C5-N4-C3	3.20	116.03	108.83
4	Z	101	MES	O3S-S-C8	2.91	110.47	105.77

For Manuscript Review

There are no chirality outliers.

All (5) torsion outliers are listed below:

Mol	Chain	Res	Type	Atoms
4	Z	101	MES	C8-C7-N4-C5
4	Z	101	MES	C7-C8-S-O3S
4	Z	101	MES	C8-C7-N4-C3
4	Z	101	MES	C7-C8-S-O1S
4	Z	101	MES	C7-C8-S-O2S

There are no ring outliers.

No monomer is involved in short contacts.

5.7 Other polymers (i)

There are no such residues in this entry.

5.8 Polymer linkage issues (i)

There are no chain breaks in this entry.

For Manuscript Review

6 Fit of model and data (i)

6.1 Protein, DNA and RNA chains (i)

In the following table, the column labelled '#RSRZ> 2' contains the number (and percentage) of RSRZ outliers, followed by percent RSRZ outliers for the chain as percentile scores relative to all X-ray entries and entries of similar resolution. The OWAB column contains the minimum, median, 95th percentile and maximum values of the occupancy-weighted average B-factor per residue. The column labelled 'Q< 0.9' lists the number of (and percentage) of residues with an average occupancy less than 0.9.

Mol	Chain	Analysed	<RSRZ>	#RSRZ>2			OWAB(Å^2)	Q<0.9
1	C	119/121 (98%)	0.30	8 (6%)	17	16	17, 26, 40, 66	0
1	M	120/121 (99%)	0.05	6 (5%)	28	29	16, 23, 34, 42	0
2	X	72/95 (75%)	0.02	4 (5%)	24	23	16, 22, 39, 66	0
2	Z	74/95 (77%)	-0.17	2 (2%)	54	55	15, 22, 37, 58	0
3	B	133/144 (92%)	-0.14	6 (4%)	33	34	15, 20, 30, 68	0
3	I	133/144 (92%)	0.27	8 (6%)	21	20	16, 22, 44, 80	0
All	All	651/720 (90%)	0.08	34 (5%)	27	26	15, 22, 39, 80	0

All (34) RSRZ outliers are listed below:

Mol	Chain	Res	Type	RSRZ
1	C	263	PHE	9.1
3	B	133	GLY	7.9
3	I	26	GLY	6.9
3	I	27	GLY	5.4
3	I	133	GLY	5.3
2	X	18	PRO	5.2
3	B	1	GLN	4.7
3	I	28	THR	4.5
2	Z	74	GLY	4.4
3	I	1	GLN	3.9
1	C	145	ALA	3.8
1	M	187	PRO	3.4
2	X	17	PRO	3.3
1	C	187	PRO	3.0
1	M	203	SER	3.0
1	M	145	ALA	2.9
2	X	19	GLY	2.8
1	M	237[A]	CYS	2.6
2	Z	50	SER	2.4

Continued on next page...

For Manuscript Review

Continued from previous page...

Mol	Chain	Res	Type	RSRZ
3	B	65	GLN	2.4
1	C	203	SER	2.3
3	B	132	GLY	2.2
1	C	216	SER	2.2
1	M	146	SER	2.2
3	B	62[A]	GLN	2.2
1	M	216	SER	2.1
1	C	182	TRP	2.1
3	B	36	TRP	2.1
3	I	62	GLN	2.1
3	I	32	TYR	2.1
3	I	2	VAL	2.1
2	X	72	ARG	2.0
1	C	188	GLY	2.0
1	C	262	TYR	2.0

6.2 Non-standard residues in protein, DNA, RNA chains (i)

There are no non-standard protein/DNA/RNA residues in this entry.

6.3 Carbohydrates (i)

There are no monosaccharides in this entry.

6.4 Ligands (i)

In the following table, the Atoms column lists the number of modelled atoms in the group and the number defined in the chemical component dictionary. The B-factors column lists the minimum, median, 95th percentile and maximum values of B factors of atoms in the group. The column labelled 'Q< 0.9' lists the number of atoms with occupancy less than 0.9.

Mol	Type	Chain	Res	Atoms	RSCC	RSR	B-factors($Å^2$)	Q<0.9
4	MES	Z	101	12/12	0.89	0.32	35,63,82,93	0

6.5 Other polymers (i)

There are no such residues in this entry.

For Manuscript Review
